# Global analysis of HBV-mediated host proteome and ubiquitylome change in HepG2.2.15 human hepatoblastoma cell line

**DOI:** 10.1186/s13578-021-00588-3

**Published:** 2021-04-17

**Authors:** Sen Yuan, Yousaf Tanzeel, Xuezhang Tian, Dandan Zheng, Naz Wajeeha, Jiaqi Xu, Yujia Ke, Zuopeng Zhang, Xiaojun Peng, Long Lu, Guihong Sun, Deyin Guo, Min Wang

**Affiliations:** 1 School of Basic Medical Sciences, Wuhan University, Wuhan, People’s Republic of China; 2 Jingjie PTM BioLab (Hangzhou) Co. Ltd., Hangzhou, People’s Republic of China; 3 School of Information Management, Wuhan University, Wuhan, People’s Republic of China; 4 Hubei Provincial Key Laboratory of Allergy and Immunology, Wuhan, People’s Republic of China; 5 School of Medicine, Sun Yat-Sen University, Shenzhen, People’s Republic of China

**Keywords:** HBV, Ubiquitination, Up-regulation, Down-regulation

## Abstract

Hepatitis B virus (HBV) infection remains a major health issue worldwide and the leading cause of cirrhosis and hepatocellular carcinoma (HCC). It has been reported previously that HBV invasion can extensively alter transcriptome, the proteome of exosomes and host cell lipid rafts. The impact of HBV on host proteins through regulating their global post-translational modifications (PTMs), however, is not well studied. Viruses have been reported to exploit cellular processes by enhancing or inhibiting the ubiquitination of specific substrates. Nevertheless, host cell physiology in terms of global proteome and ubiquitylome has not been addressed yet. Here by using HBV-integrated HepG2.2.15 model cell line we first report that HBV significantly modify the host global ubiquitylome. As currently the most widely used HBV cell culture model, HepG2.2.15 can be cultivated for multiple generations for protein labeling, and can replicate HBV, express HBV proteins and secrete complete HBV Dane particles, which makes it a suitable cell line for ubiquitylome analysis to study HBV replication, hepatocyte immune response and HBV-related HCC progression. Our previous experimental results showed that the total ubiquitination level of HepG2.2.15 cell line was significantly higher than that of the corresponding parental HepG2 cell line. By performing a Ubiscan quantification analysis based on stable isotope labeling of amino acids in cell culture (SILAC) of HepG2.2.15 and HepG2 cell lines, we identified a total of 7188 proteins and the protein levels of nearly 19% of them were changed over 2-folds. We further identified 3798 ubiquitinated Lys sites in 1476 host proteins with altered ubiquitination in response to HBV. Our results also showed that the global proteome and ubiquitylome were negatively correlated, indicating that ubiquitination might be involved in the degradation of host proteins upon HBV integration. We first demonstrated the ubiquitination change of VAMP3, VAMP8, DNAJB6, RAB8A, LYN, VDAC2, OTULIN, SLC1A4, SLC1A5, HGS and TOLLIP. In addition, we described 5 novel host factors SLC1A4, SLC1A5, EIF4A1, TOLLIP and BRCC36 that efficiently reduced the amounts of secreted HBsAg and HBeAg. Overall, the HBV-mediated host proteome and ubiquitylome change we reported will provide a valuable resource for further investigation of HBV pathogenesis and host-virus interaction networks.

**Supplementary Information:**

The online version contains supplementary material available at 10.1186/s13578-021-00588-3.

## Introduction

Hepatitis B virus (HBV) infection is a worldwide life-threatening health problem that leads to chronic and acute hepatitis. Although more than 50 years have passed since the discovery of HBV and effective vaccine has being used against it for about 30 years [1], a serious health issue still remains as it is estimated that approximately 350 million people worldwide have been affected by chronic infection of HBV, the leading cause of liver diseases cirrhosis and hepatocellular carcinoma [2–4]. HBV belongs to *Hepadnaviridae* family of small enveloped DNA virus that replicates primarily in the livers of their hosts and exhibits similarities to retrovirus i.e., it replicates through reverse transcription of an RNA intermediate. There is the interaction between numerous host cell factors and HBV proteins, which governs HBV replication in hepatocytes and plays an important role in the development of HBV-associated HCC [5, 6].

Ubiquitination is a highly dynamic post-translational modification (PTM) process which plays vital role in protein degradation, signal transduction, endocytosis, DNA replication and repair mediating via proteasomal dependent or independent pathways which enables constant proteome variations according to cell surroundings [7, 8]. This process involves the covalent attachment of the C-terminal carboxyl group of ubiquitin, a small highly conserved protein of 76 amino acids in all eukaryotes. Usually, ubiquitin protein is also known as **death mark** as its main role in degradation [9]. As ubiquitin itself contains seven internal lysine residues, poly-ubiquitin chains formation takes place on a target protein by consecutive rounds of secondary ubiquitin molecules linked to lysine residues of the previous ubiquitin molecule, e.g., K48-linked chains are critical for protein degradation while other polyubiquitinations (e.g., on K63, K33, K29, K27, K11, K6, and M1) and monoubiquitinations contributed in processes such as endocytic trafficking, NF-κB pathway, translation and DNA repair [10–12]. During viral infection, the ubiquitin system is an important part of the cellular defense mechanism and viruses exploit it for their own advantage. In the last two decades, many research studies have shown that ubiquitin–proteasome system (UPS) interacts with the replication of major human pathogens such as herpesviruses, poxviruses, hepadnaviruses, adenoviruses, influenza viruses, retroviruses, coronaviruses, paramyxoviruses, picornaviruses, and rotaviruses. In all these cases, specific viral proteins, i.e., transcriptional trans-activators, are able to interact with the ubiquitin-conjugating machinery, leading to an increase in viral gene expression by down-regulation of the NF-κB and/or IFN production [13, 14]. In the recent studies, it has shown that HBV exploits the host immune responses by up-regulation or down-regulation of the host cell proteins as a result in its enhanced propagation, release and surpass host immune system. For example, HBV modulates not only various cell-cycle regulators, including p53,CDK1, CyclinD, CyclinE, p21, and CDK2, but also many cellular signal transduction pathways such as the PI3K/AKT, the mTOR, and the MAPK pathways [15–18].

Recently, HBV-mediated transcriptome-wide alterations in gene expression were studied to understand host-virus interactions [16]. HBc was shown to promote the expression of metabolic enzymes and the secretion of metabolites in HCC cells in a combined proteomics and metabolomics approach [19]. Considering that exosomes act as important mediators in cell–cell communication and viruses manipulate the infection process by secretion of specific viral and cellular components to exosomes, label-free and SILAC-based quantitative proteomic analyses of exosome proteins have been employed to study exosome content changes induced by HBx overexpression and HBV replication [20, 21]. It has been reported that some host cell proteins, such as tumor suppressor p53, Id-1, X-linked tumor suppressor TSPX, Hdj1, and phospholipid scramblase 1(PLSCR1), limit HBV replication by repressing the expression of HBx via the ubiquitin–proteasome system (UPS) [22]. However, there is no investigation conducted regarding the overall manipulation of host ubiquitylome and proteome by HBV integration. In this study, we performed a Ubiscan quantification analysis based on stable isotope labeling of amino acids in cell culture (SILAC) to study the host ubiquitylome and proteome in HepG2.2.15 and HepG2, the HBV model cell line that stably producing HBV virus and its corresponding parental HepG2 cell line.

## Materials and methods

### Cell culture and reagents

HepG2.2.15 and HepG2 cells (ATCC, Rockville, MD, USA) were maintained in Dulbecco’s modified Eagle’s medium (DMEM, Gibco, USA) containing 10% dialyzed FBS (Gibco, USA), penicillin (107 U/L) and streptomycin (10 mg/L) at 37 °C in a humidified atmosphere containing 5% CO_2_. Chemicals purchased from Sigma-Aldrich (St. Louis, MO, USA) were DMSO, iodoacetamide (IAA), formic acid (FA), trifluoroacetic acid (TFA) and dithiothreitol (DTT). SILAC™ Protein Identification and Quantitation Media Kit purchased from Thermo Scientific (Agawam, MA, USA). 2-D Quant kit was purchased from GE Healthcare. Anti-hemagglutinin (anti-HA) and anti-Flag antibodies were obtained from ABclonal (Woburn, MA, USA).

### SILAC labeling

The HepG2-K6R10 cells were labeled with the l-^13^C_6_-lysine/l-^13^C_6_^15^N_4_-arginine, whereas the HepG2.2.15-K0R0 cells were labeled with the l-^12^C_6_-lysine/l-^12^C_6_^14^N_4_-arginine separately using a SILAC Protein Quantitation Kit according to manufacturer’s instructions. Briefly, the cell line was grown in DMEM medium supplemented with 10% fetal bovine serum and either the “heavy” form of [^13^C_6_]-l-lysine/[^13^C_6_^15^N_4_]-l-arginine or “light” form [^12^C_6_]-l-lysine/[^12^C_6_^14^N_4_]-l-arginine for more than 7 generations before being harvested, to achieve more than 97% labeling efficiency.

### Protein extraction and in-solution trypsin digestion

We performed protein extraction and trypsin digestion according to previously described protocols [23]. The harvested “heavy” and “light” labeled cells were sonicated 3 times on ice using a high intensity ultrasonic processor (Scientz, Ningbo, China) in lysis buffer (8 M Urea, 5 mM DTT, 2 mM EDTA, 1.0% cocktail III and 50 μM PR619). The remaining debris was removed by centrifugation at 20,000×*g* at 4 °C for 10 min. After concentration measurement, equal amounts of crude proteins in the supernatant labeled. “Heavy” or “light” was mixed and the crude proteins were precipitated with TFA using a 15% final concentration (v/v) (soluble fraction). After washing twice with − 20 °C acetone, the protein pellets were dissolved in 100 mM NH_4_HCO_3_ (pH 8.0) for trypsin digestion. Trypsin solution (Promega) (trypsin: protein = 1:50) was added to proteins and then the protein pellets were digested at 37 °C for 16 h. After alkylation reaction, trypsin (trypsin: protein = 1:100) was added again and incubated (37 °C, 4 h).

### HPLC fractionation

The sample was then fractionated into fractions by high pH reverse-phase HPLC using Agilent 300Extend C18 column (5 μm particles, 4.6 mm ID, 250 mm length). Briefly, peptides were first separated with a gradient of 2% to 60% acetonitrile in 10 mM ammonium bicarbonate pH 10 over 80 min into 80 fractions. Then, the peptides were combined into 6 fractions and dried by vacuum centrifuging.

### Affinity enrichment

To enrich ubiquitinated peptides, tryptic peptides dissolved in NETN buffer (100 mM NaCl, 1 mM EDTA, 50 mM Tris–HCl, 0.5% NP-40, pH 8.0) were incubated with pre-washed antibody beads (PTM Biolabs) at 4 °C overnight with gentle shaking. The beads were washed four times with NETN buffer and twice with ddH_2_O. The bound peptides were eluted from the beads with 0.1% TFA. The eluted fractions were combined and vacuum-dried. The resulting peptides were cleaned with C18 ZipTips (Millipore, USA) according to the manufacturer’s instructions, followed by LC–MS/MS analysis.

### LC–MS/MS analysis

We performed LC–MS/MS analysis according to previously described protocols [23]. Briefly, Peptides were dissolved in 0.1% FA and directly loaded onto a reversed-phase pre-column (Acclaim PepMap 100, Thermo Scientific). To separate peptide fractions, a reversed-phase analytical column (Acclaim PepMap RSLC, Thermo Scientific) was used, with gradient of an increase in solvent B (0.1% FA in 98% ACN) from 6 to 22% for 26 min, 22% to 35% for 8 min, went up to 80% in 3 min and staying at 80% for another 3 min. The whole process happened at a constant flow rate of 300 nL/min on an EASY-nLC 1000 UPLC system. The following analysis of resulting peptides was carried out by Q Exactive™ Plus hybrid quadrupole-Orbitrap mass spectrometer (ThermoFisher Scientific).

### Database search

The resulting MS/MS data were processed using MaxQuant with integrated Andromeda search engine (v.1.5.2.8). Tandem mass spectra were searched against Swiss-Prot human database concatenated with reverse decoy database.

For proteomic peptides, Trypsin/P was specified as cleavage enzyme allowing up to 2 missing cleavages, 5 modifications per peptide and 5 charges. Mass error was set to 10 ppm for precursor ions and 0.02 Da for fragment ions. Carbamidomethylation on Cys was specified as fixed modification, oxidation on Met and acetylation on protein N-terminal were specified as variable modifications. False discovery rate (FDR) thresholds for protein, peptide and modification site were specified at 1%. Minimum peptide length was set at 7. All the other parameters in MaxQuant were set to default values.

For peptides with ubiquitinated Lys sites, Trypsin/P was SPECIFIED as cleavage enzyme allowing up to 4 missing cleavages, 5 modifications per peptide and 5 charges. Mass error was set to 10 ppm for precursor ions and 0.02 Da for fragment ions. Carbamidomethylation on Cys was specified as fixed modification and oxidation on Met, GlyGly on Lysine and oxidation on Met were specified as variable modifications. False discovery rate (FDR) thresholds for protein, peptide and modification site were specified at 1%. Minimum peptide length was set at 7. All the other parameters in MaxQuant were set to default values. The site localization probability was set as ≥ 0.75.

### Bioinformatics analysis

GO annotation was analyzed through the UniProt-GOA database and InterProScan software. WoLF PSORT was applied to predict subcellular localization [24]. We utilized Kyoto Encyclopedia of Genes and Genomes (KEGG) database for protein pathways analysis. The KEGG online service tool KAAS was used to annotate the proteins’ KEGG database descriptions. The annotation results were mapped on the KEGG pathway database using the KEGG online service tool KEGG mapper. CORUM database was used to annotate protein complexes, InterProScan domain database for protein domain annotation and Motif-x for motif analysis. Heatmap was drawn by the “heatmap.2” of R-package. The search tool for the Retrieval of Interacting Genes/Proteins (STRING) database was used to outline protein–protein interactions. Two-tailed Fisher’s exact test was applied**.**

### Enzyme-linked immune-sorbent assay

The hepatitis B surface antigen (HBsAg) and hepatitis B e antigen (HBeAg) viral-secreted protein levels in the supernatant were determined using enzyme-linked immunosorbent assay (ELISA) kits (Kehua Bio-engineering, Shanghai, China).

### Real-time quantitative PCR

Total RNA, including miRNAs, and protein were prepared from cells or tissues. RNA was reverse-transcribed using the M-MLV Reverse Transcriptase Kit (Invitrogen; Thermo Fisher Scientific, Waltham, MA). Real-time quantitative PCR was performed using the SYBR Select Master Mix (Life Technologies, Carlsbad, CA).

### Immunoprecipitation and Western blotting

For detection of ubiquitination, we performed immunoprecipitation according to previously described protocols [25]. Cells were cultured in 60-mm dish and treated with 10 μM MG132 for 2–4 h. Cells were lysed after 48 h with Lysis Buffer (50 mM Tris–Cl [pH 8.0], 150 mM NaCl, 0.1% SDS, 1% NP-40, 0.5% Sodium deoxycholate) supplemented with 1% cocktail and freshly dissolved 10 mM *N*-ethylmaleimide (NEM). After centrifugation, the supernatant was immunoprecipitated with Flag-antibodies and protein A/G beads then the mixture was incubated overnight at 4 °C. The beads were washed 3 times with washing buffer (20 mM Tris–Cl [pH 7.5], 150 mM NaCl, 0.5% NP-40 mM ethylene diamine tetraacetic acid). The proteins bound to the beads were analyzed by western blot with anti-Ub-antibodies.

### Statistical analysis

All statistical analyses were performed using GraphPad Prism 6 software. Data are presented as the mean ± standard deviation (SD), and the two-tailed Student’s t-test (two-sample equal variance) was used to measure the significance of observed differences between two groups, with *P* < 0.05 considered to be statistically significant. In all cases, at least three independent experiments were performed. NS, no significant difference. *P < 0.05, **P < 0.01, ***P < 0.001.

## Results

### HBV changes proteome profile in HepG2.2.15 cell line

**Fig. 1:**
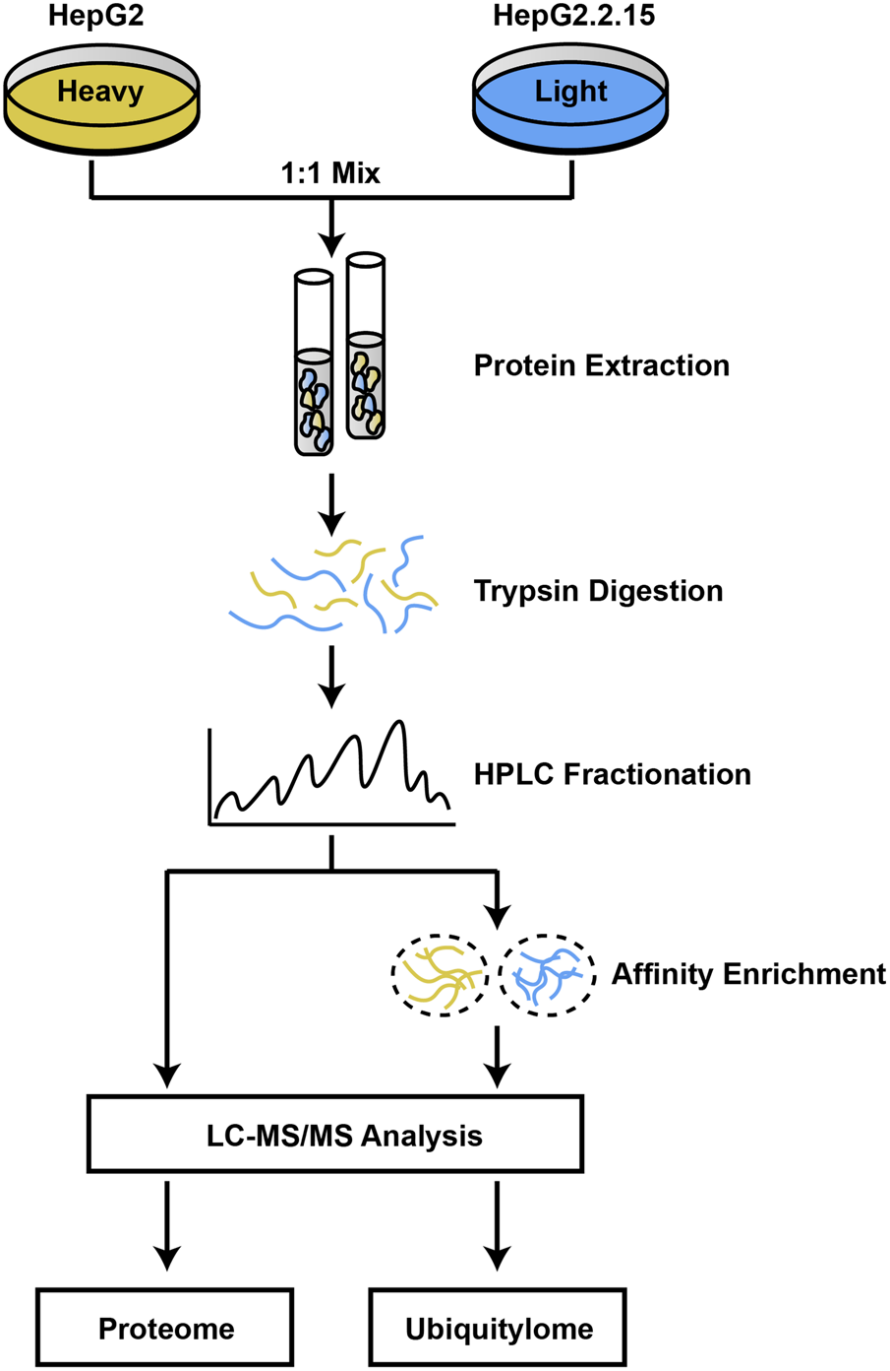
The systematic workflow for quantitative profiling of global proteome, ubiquitylome in HepG2 and HepG2.2.15 cells with and without HBV integration

**Fig. 2:**
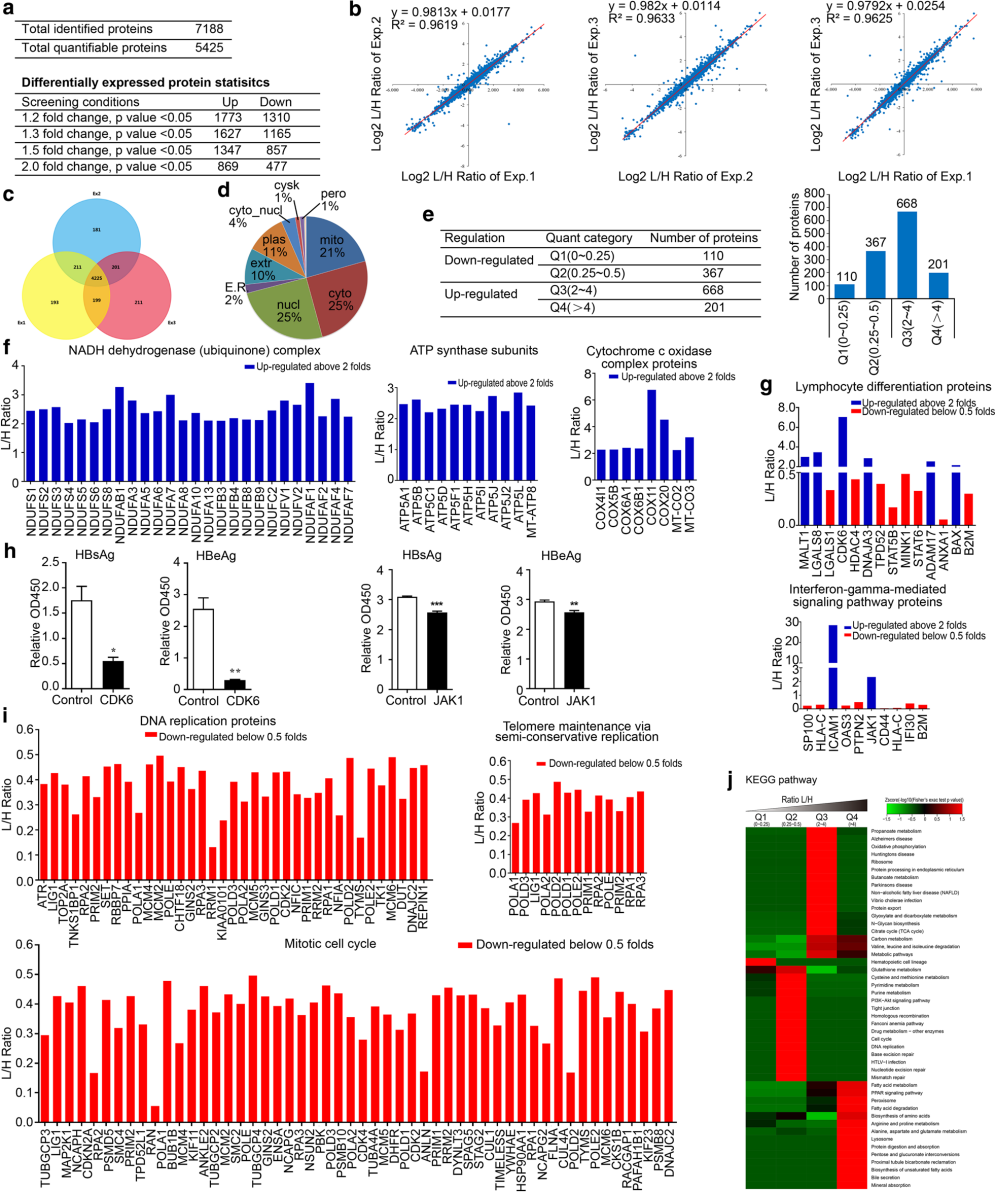
Host proteome upon HBV integration. **a** Summary of identified and quantified proteins and peptides. **b** Pearson’s correlation (R) plots for three representative experiments from HepG2.2.15 cells. **c** Venn diagrams of peptides in HepG2.2.15 Peptide numbers are indicated. **d** The subcellular location of differentially expressed proteins. **e** Distribution of regulated proteins quantification results. **f**, **g** L/H ratios for selected regulated proteins from three biological replicates in HepG2.2.15 cells. **h** The levels of the secreted HBeAg and HBsAg were determined by ELISA from the cell culture supernatants samples co-transfected (1:1) with pHBV1.3 and indicated plasmids. The data represent the average of three independent experiments and were analyzed with a two-tailed unpaired t test. Graphs indicate mean ± S.D. (n = 3) derived from three independent experiments. *P < 0.05, **P < 0.01, ***P < 0.001, ****P < 0.0001. **i** L/H ratios for selected regulated proteins from three biological replicates in HepG2.2.15 cells. **j** KEGG pathway analysis

To quantitatively profile the changes in the cellular ubiquitylome and proteome in response to HBV integration and replication, we employed the Ubiscan technology which combines Ub-antibody-based peptide enrichment with liquid chromatography-tandem mass spectrometry (LC–MS/MS) to quantitatively profile ubiquitination modifications. HepG2.2.15 cells which stably produces HBV virus and the corresponding parental HepG2 cells were labeled by amino acids in cell culture, followed by HPLC fractionation, antibody affinity enrichment and SILAC-based mass spectrometry (MS) analysis (Fig. 1). Bioinformatics analysis for systematic quantification of proteome and lysine ubiquitylome was performed based on three paired biological replicates. A total of 7188 proteins from the whole proteome were identified, among which a relatively large number of 5425 proteins were quantified (Additional file 5: Table S1), compared to previous reported proteomic analysis of exosomes secreted from HBV-invaded cells [20, 21]. Across these quantified proteins, 1346 were changed over twofolds (869 up-regulated with light:heavy [L:H] ratios ≥ 2.0 and 477 down-regulated with light:heavy [L:H] ratios ≥ 0.5), 2204 were changed over 1.5-folds (1347 up-regulated and 857 down-regulated), 2792 were changed over 1.3-folds (1627 up-regulated and 1165 down-regulated) and 3083 were changed over 1.2-folds (1773 up-regulated and 1310 down-regulated) (Fig. 2a). Pearson’s correlation coefficients of up to 0.96 indicated high reproducibility among all three biological replicates (Fig. 2b), concomitant with significant overlap across biological triplicates for each experiment (Fig. 2c). All differentially expressed proteins were divided into four quantiles (Q1–Q4) according to their light: heavy [L:H] ratios Q1 (0 < 0.25), Q2 (0.25 < 0.5), Q3 (2 < 4), Q4 (> 4) (Fig. 2e).

Gene ontology annotation was applied to classify proteins in terms of their subcellular localization (Fig. 2d). According to the sub-cellular localization analysis, we found that the up-regulated proteins were highly enriched in mitochondria 29%, cytoplasm 19% and nucleus 18% (Additional file 1: Figure S1A), whereas down-regulated proteins were enriched in nucleus 39%, cytoplasm 36% (Additional file 1: Figure S1B), indicating that HBV promotes the up-regulation of proteins in mitochondria, plasma membranes and peroxisomes, and the down-regulation of proteins in the nucleus and cytoplasm. Gene ontology (GO) enrichment-based clustering analysis (Additional file 1: Figure S1C) also showed that up-regulated proteins have mainly mitochondria and membrane related functions such as oxidoreductase activity, NADH dehydrogenase activity and ion transmembrane transporter activity; whereas down-regulated proteins have mainly DNA replication related functions. In the cellular component category, the up-regulated proteins were highly enriched in mitochondria, ribosomes and membrane region while the down-regulated proteins were enriched in cytosol, nuclear replisome and cytoskeleton (Additional file 1: Figure S1D). In biological process analysis (Additional file 1: Figure S1E), proteins which were involved in cellular respiration NADH dehydrogenase ubiquinone complex subunits NDUFB9, NDUFA13, NDUFB3, NDUFB8, ATP synthase complex proteins ATP5O, ATP5C1, ATP5H, ATP5I, ATP5J2, cytochrome c oxidase complex component proteins COX4I1, COX5B, COX6A1, MT-CO2, and MT-CO3 were highly up-regulated (Fig. 2f). The analysis of molecular functions (Additional file 1: Figure S1F) showed that oxidoreductase activity, NADH dehydrogenase activity and ion transmembrane transporter activity were enriched upon HBV integration while DNA polymerase activity, enzyme regulator activity and phosphatase regulation activity were down-regulated. The KEGG pathway enrichment analysis (Fig. 2j; Additional file 2: Figure S2A) showed that the oxidative, metabolic and PPAR signaling pathways were enriched in response to HBV integration (Additional file 2: Figure S2B), in concordance with the previous proteomic and metabolomic results that HBc may accelerate the processes of cell metabolism by up-regulating proteins associated with the alteration of metabolic pathways [19]. DNA replication, mismatch repair, pyrimidine metabolism, biosynthesis of amino acids and p53 signaling pathways were down-regulated. Protein complex enrichment analysis revealed that 55S, 39S and 28S ribosomal subunits (mitochondrial) were enriched. DNA synthesome complex and replication complex (RC) during S-phase of cell cycle were distinctly down-regulated (Additional file 2: Figure S2C). Further, enrichment analysis of protein domains demonstrated that thioredoxin fold (NDUFV2, TMX2, TXN2) and NAD(P)-binding domain (H6PD, FDXR) were highly up-regulated (Additional file 2: Figure S2D). Redox-active proteins possess thioredoxin-like fold that exhibits a role in lipid, glucose metabolism and cardiac function. Their elevated levels are associated with oxidative stress in numerous cancers [26], therefore, act as a biomarker in hepatitis B and C virus-induced liver carcinogenesis [27].

We tested the effect of two up-regulated proteins on HBV replication, including CDK6 and JAK1 which are involved in lymphocyte differentiation and interferon-gamma-mediated signaling pathway respectively (Fig. 2g). Interestingly, ectopic expression of CDK6 and JAK1 significantly reduced the amounts of secreted HBsAg, HBeAg and total RNA (Fig. 2h), suggesting that CDK6 and JAK1 may be up-regulated by host cell to defense HBV invasion. The up-regulation of CDK6 upon HBV invasion was previously showed in a multi-omics based studies [19]. On the other hand, our results showed that proteins involved in DNA replication such as ATR, MCM2-6, DNA ligase 1, DNA polymerase subunits POLD1-2, POLE2, CDK2, PCNA-associated factor KIAA0101, mitotic cell cycle CDKN2A, TPD52L1 MAP2K1, PSMD5, HSP90AA1 and telomere maintenance proteins POLA1-2, POLD2 were all down-regulated (Fig. 2i). Among them, ATR, CDK2, CDKN2A have been previously reported to be deregulated and associated with the cell cycle in HBV-infected primary human hepatocytes (PHHs) to render a cellular environment that is favorable for productive HBV infection [28]. Therefore, the proteome profile modifications, including both up-regulation and down-regulation of host proteins, could be the response of host cells to fight against HBV invasion.

### HBV integration changes ubiquitylome profile in HepG2.2.15 cell line

**Fig. 3:**
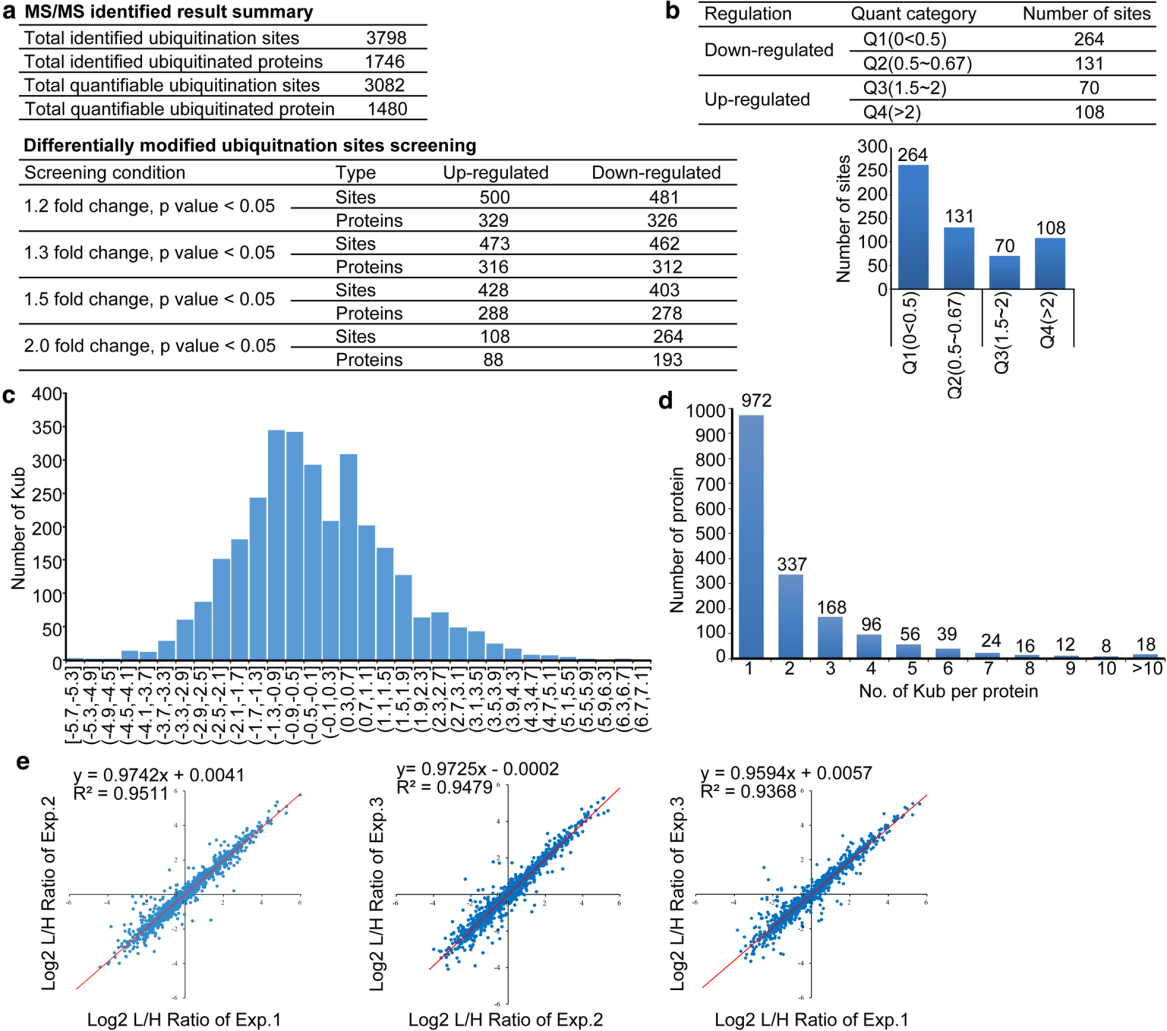
Host ubiquitylome upon HBV integration. **a** Summary of identified and quantified sites and proteins. **b**, **c** Distribution of regulated Kub-sites quantification results. **d** Association between number of proteins and number of Kub-sites per protein is shown. **e** Pearson’s correlation (R) plots for three representative experiments from HepG2.2.15 cells

We quantified a total of 3798 ubiquitinated Lys sites in 1476 proteins (Fig. 3a). Among these ubiquitinated Lys sites, 372 were changed over twofolds including 108 up-regulated and 264 down-regulated (Fig. 3b, c). There are 972 proteins whose ubiquitination changes at a single lysine position. 18 proteins were found to be ubiquitinated at more than 10 lysine sites (Fig. 3d). The significant overlap across biological triplicates and Pearson’s correlation coefficients of up to 0.94 represented close imitation among biological triplicate samples (Fig. 3e and Additional file 3: Figure S3A).

**Fig. 4:**
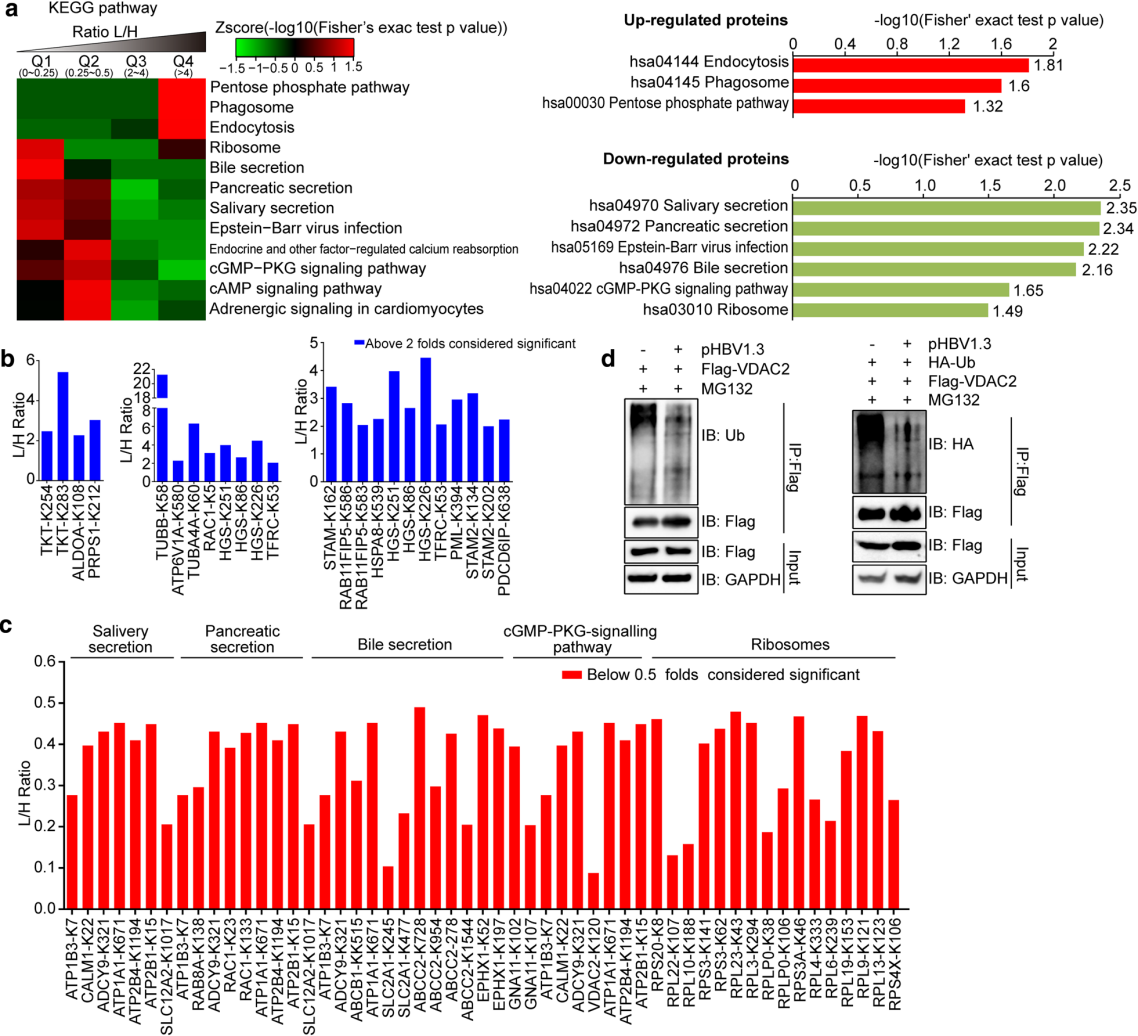
Enrichment of KEGG pathway analysis for the quantified ubiquitylome. **a** KEGG pathway analysis. **b**, **c** L/H ratios for selected regulated Kub-sites & proteins from three biological replicates in HepG2.2.15 cells. **d** Co-immunoprecipitation and immunoblot analysis of extracts of Huh7 cells transfected with various combinations of plasmid encoding FLAG-tagged VDAC2 and HA-tagged wild type ubiquitin, Hepatitis B virus (HBV) 1.3-fold genome plasmid (pHBV1.3) and treated with MG132, are shown

To identify metabolic pathways related to proteins with ubiquitination change in response to HBV integration, we performed KEGG database analysis (Fig. 4a). A number of vital pathways, including pentose phosphate pathway (TKT-K254, TKT-K283, ALDOA-K108), phagosome (TUBB-K58, TUBA4A-K60, RAC1-K5, HGS-K86, HGS-K226, HGS-K251) and endocytosis (STAM-K162, STAM2-K134, STAM2-K202, HSPA8-K539) were enriched among proteins with increased ubiquitination in response to HBV integration (Fig. 4b). Ubiquitination of salivary, pancreatic, bile secretion pathway (ATP1A1-K671, RAB8A-K138, SLC12A2-K1017) and cGMP-PKG signaling pathway (VDAC2-K120, CALM1-K22, ADCY9-K321) were found to be down-regulated (Fig. 4c). Ubiquitination down-regulation of exogenous VDAC2 induced by HBV was confirmed by immunoblotting (Fig. 4d). Similarly, VDAC1 and VDAC3 were found to be up-regulated in a lipid raft based proteomic analysis [29].

**Fig. 5:**
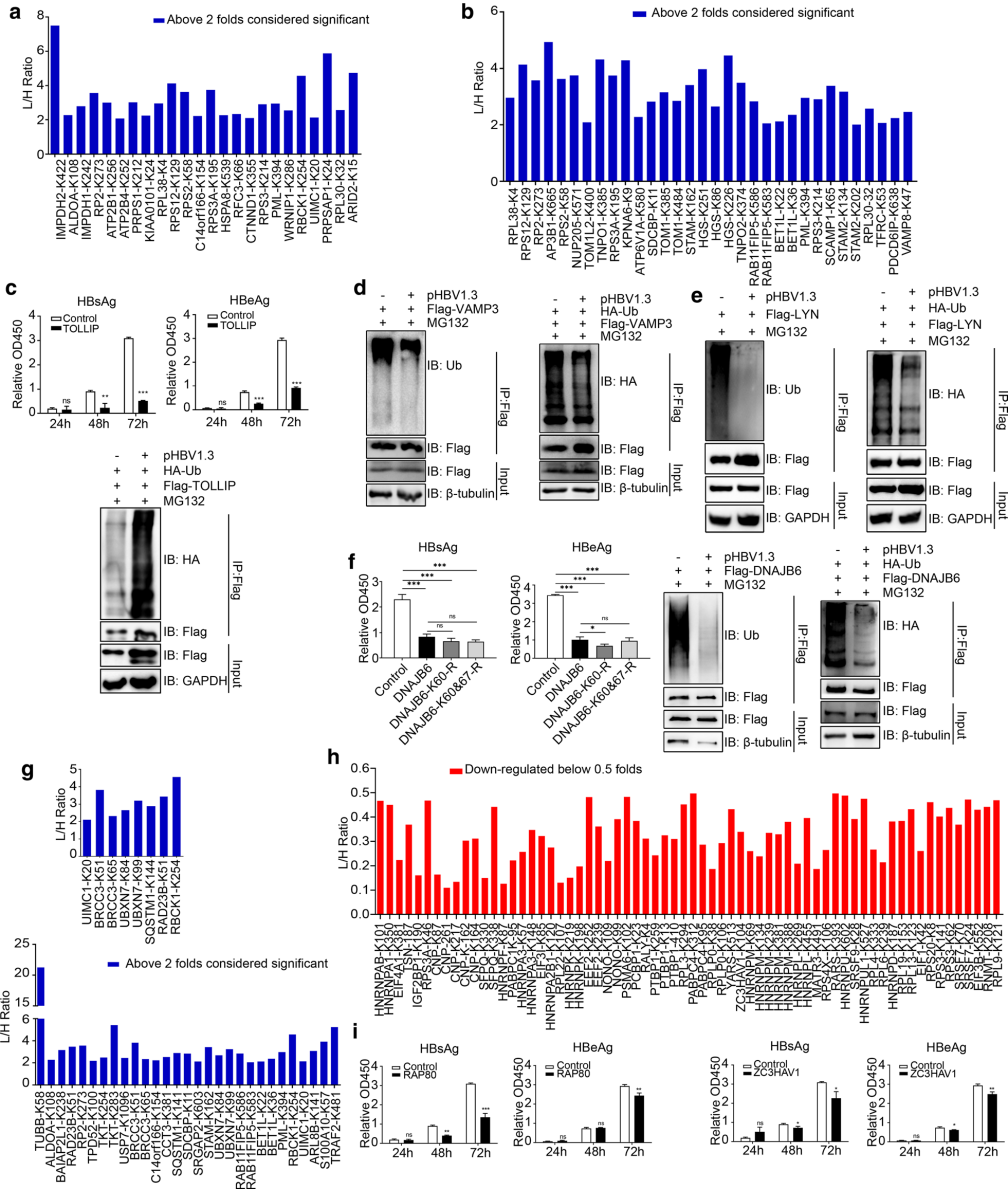
Functional enrichment-based clustering analysis for the quantified ubiquitylome. **a**, **b** L/H ratios for selected regulated Kub-sites & proteins from three biological replicates in HepG2.2.15 cells. **c** The levels of the secreted HBeAg and HBsAg were determined by ELISA from the cell culture supernatants samples co-transfected (1:1) with pHBV1.3 and flag-tagged TOLLIP. Co-immunoprecipitation and immunoblot analysis of extracts of Huh7 cells transfected with FLAG-tagged TOLLIP and HA-tagged wild type ubiquitin, Hepatitis B virus (HBV) 1.3-fold genome plasmid (pHBV1.3) and treated with MG132, are shown. **d**, **e** Co-immunoprecipitation and immunoblot analysis of Huh7 cells transfected with FLAG-tagged indicated plasmids together with HA-tagged wild type ubiquitin, Hepatitis B virus (HBV) 1.3-fold genome plasmid (pHBV1.3) and treated with MG132, are shown. **f** The levels of the secreted HBeAg and HBsAg were determined by ELISA from the cell culture supernatants samples co-transfected (1:1) with pHBV1.3 and flag-taged DNAJB6. Co-immunoprecipitation and immunoblot analysis of extracts of Huh7 cells transfected with FLAG-tagged DNAJB6 and HA-tagged wild type ubiquitin, Hepatitis B virus (HBV) 1.3-fold genome plasmid (pHBV1.3) and treated with MG132, are shown. **g**, **h** L/H ratios for selected regulated Kub-sites & proteins from three biological replicates in HepG2.2.15 cells. **i** The levels of the secreted HBeAg and HBsAg were determined by ELISA from the cell culture supernatants samples co-transfected (1:1) with pHBV1.3 and indicated plasmids. The data represent the average of three independent experiments and were analyzed with a two-tailed unpaired t test. Graphs indicate mean ± S.D. (n = 3) derived from three independent experiments. *P < 0.05, **P < 0.01, ***P < 0.001, ****P < 0.0001

To identify biological processes related to proteins with ubiquitination change in response to HBV integration, we performed enrichment-based clustering analyses **(**Additional file 3: Figure S3B, S3C). The proteins related to nucleoside metabolism, biosynthetic process (Fig. 5a) (ALDOA-K108, RP2-K273, IMPDH2-K422, IMPDH1-K242, KIAA0101-K24) and protein transport (TOM1-K385, TOM1-K484, STAM-K162, STAM2-K134, STAM2-K202) (Fig. 5b) were highly ubiquitinated. By contrast, ubiquitination of RNA metabolic process (EIF4A1-K381, SET-K167, POLR2B-K146), viral process (RAC1-K123, RAC1-K133, USP7-K869, CBX5-K42, CBX5-K91) and secretion (SLC9A3R1-50, SLC9A3R1-K101, SLC29A1-K239, VAMP3-K66, LYN-K20, COPA-K238) were down-regulated (Additional file 3: Figure S3D).

To further prove the reliability of our UbiScan results, we picked several more proteins which have not previously reported to be ubiquitinated upon HBV infection but showed significant ubiquitination change in our results. Consistent with the UbiScan results (Additional file 5: Table S1), HBV transfection considerably enhanced ubiquitination of exogenous toll-interacting protein (TOLLIP) (Fig. 5c, right), suggesting that HBV may regulate its protein level through regulating its ubiquitination. Interestingly, over-expression of TOLLIP suppressed the HBV protein expression (Fig. 5c, left), implying the complicated interplay between HBV and host TOLLIP protein. We further affirmed the ubiquitination down-regulation of proteins involved in secretion, VAMP3, and tyrosine-protein kinase (LYN) in response to HBV replication (Fig. 5d, e). Although most of the proteins we chose to examine have reconfirmed our UbiScan results, there were few proteins failed to show ubiquitination or changed ubiquitination after HBV transfection. Furthermore, we experimentally confirmed ubiquitination of another protein DNAJB6 and it was in consistent with our UbiScan results (Fig. 5f, right). Over-expression of DNAJB6 significantly reduced the amounts of secreted HBsAg and HBeAg and this inhibition was independent of its lysine mutants K60R and K67R (Fig. 5f). We concluded that Ubiscan method conveyed us relatively reliable information regarding the global proteome and ubiquitylome of cells, but it is not a complete and absolute a scanning method.

The analysis of molecular functions (Additional file 3: Figure S3E) showed that ubiquitination of protein binding (TUBB-K58, TPD52-K100, USP7-K1096, TRAF2-K481, TKT-K254, TKT-K283), small conjugated and ubiquitin-binding proteins (UBXN7-K84, UBXN7-K99, RAD23B-K51, BRCC3-K51, BRCC3-K65, RBCK1-K254) were highly enriched (Fig. 5g). RNA binding proteins (HNRNPAB-K101, HNRNPA1-K350, EIF4A1-K381, ZC3HAV1-K401) were significantly down-regulated (Fig. 5h), which is consistent with the results of proteomic studies conducted on exosomes of HBV-invaded cells that HNRNPA1 was significantly increased upon HBx overexpression [21]. These proteins contain protein/RNA binding motif, thus possibly playing roles during HBV and host interaction. Such interactions could be important since ectopic expression of ZC3HAV1 and UMIC1 (RAP80) effectively inhibited HBV propagation (Fig. 5i).

**Fig. 6:**
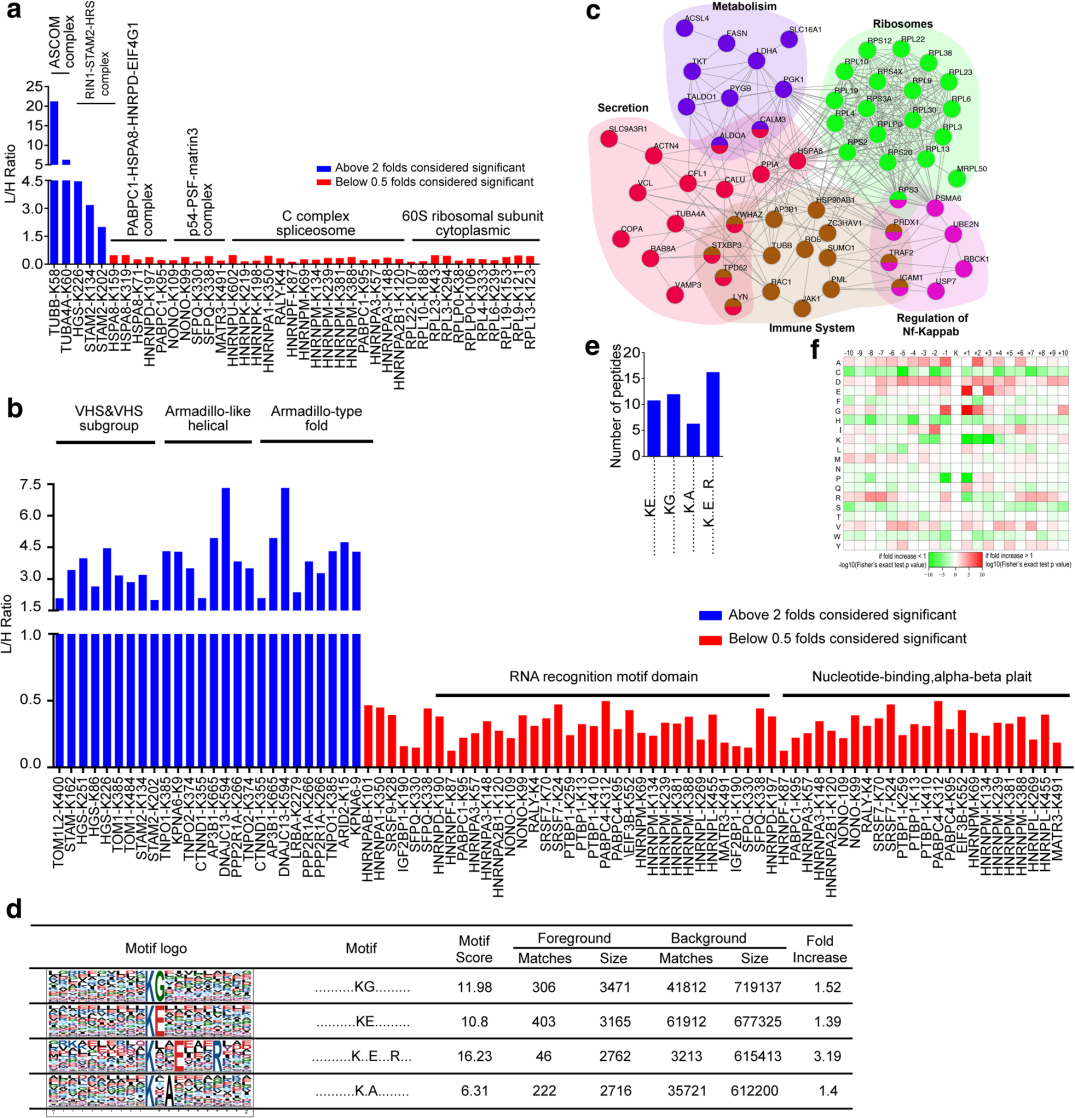
Motif analysis of all the identified Kub sites and protein–protein interaction of ubiquitinated proteins in HepG2.2.15 cells. **a**, **b** L/H ratios for selected regulated Kub-sites & proteins from three biological replicates in HepG2.2.15 cells. **c** Associations among proteins with regulated Kub-sites based on STRING and visualized in Cytoscope. **d** Ubiquitination motifs and the conservation of Kub-sites. The height of each letter corresponds to the frequency of that amino acid residue in that position. The central K refers to the ubiquitinated Lys. **e** Number of identified peptides containing ubiquitinated Lys in each motif. **f** Amino acid sequence properties of ubiquitylation sites. The heat map shows significant position-specific underrepresentation or overrepresentation of amino acids flanking the modification sites

We discovered ubiquitination of two protein complexes, ASCOM (TUBB TUBA4A) and RIN1-STAM2-HRS (HGS, STAM2) were immensely augmented. Ubiquitination of PABPC1-HSPA8-HNRPD-EIF4G1 complex (HSPA8, HNRNPD, PABPC1), C complex spliceosome complex (HNRNPU, HNRNPK, HNRNPA1, PABPC1, RALY) and ribosome 60S ribosomal subunits (RPL13, RPL10, RPL23, RPL9, RPLP0) were significantly down-regulated (Fig. 6a and Additional file 4: Figure S4A). Protein domain enrichment analysis exhibited that VHS (STAM, STAM2, HGS, TOM1), VHS subgroup (STAM, STAM2, TOM1, HGS) and armadillo-like helical (TNPO1, DNAJC13, PPP2R1A, TNPO2) domain’s ubiquitination were markedly increased while ubiquitination of RNA recognition motif domain (HNRNPAB, HNRNPA1) and nucleotide-binding, alpha–beta plait (EIF3B, HNRNPM, HNRNPL) were distinctly decreased (Fig. 6b and Additional file 4: Figure S4B). Protein–protein interaction network of the ubiquitylated proteins was established by using Cytoscape software, which revealed enrichment of components of metabolism, ribosomes, secretion, immune system and regulation of NF-κB (Fig. 6c).

### Motif analysis of ubiquitylome upon HBV integration

To further understand the properties of the identified Lys sites that were ubiquitinated, motif analysis was performed by using ubiquitin remnant motif antibody K-ε-GG against all the identified ubiquitin remnant peptides in HepG2.215 cell line. We used the Motif-X program to compare the position-specific frequencies of the amino acid residues surrounding all ubiquitinated Lys residues (Fig. 6d). Among 3798 ubiquitinated-Lys-containing peptides in 1746 proteins, we observed 4 types of conserved motifs for 987 unique sites that were prominently enriched with different abundances: xxxxxxxxxx**KG**xxxxxxxxx, xxxxxxxxxx**KE**xxxxxxxxx, xxxxxxxxxx**K**xx**E**xxx**R**xxx and xxxxxxxxxx**K**x**A**xxxxxxxx. They accounted for approximately 25% of all identified peptides (Fig. 6e), with the xxxxxxxxxx**K**xx**E**xxx**R**xxx motif exhibiting the highest score (Fig. 6f). Analysis of these motifs revealed that four distinct residues were found downstream of the ubiquitinated Lys, including glycine (G), glutamic acid (E), arginine (R) and neutral alanine (A). We examined the frequencies of neighboring amino acid residues for ubiquitinated Lys residues using iceLogo [30] and observed a significant abundance for residues such as glycine (G) and glutamic acid (E) at positions adjacent to ubiquitinated Lys residues (Gly + 1, + 2, − 1, and Glu + 1, + 3). We speculate that these conserved ubiquitination motifs could be recognized by specific E3 ligases, leading to ubiquitination of target proteins and the consequent activity regulation.

### Interaction between the global proteome and ubiquitylome

**Figure7:**
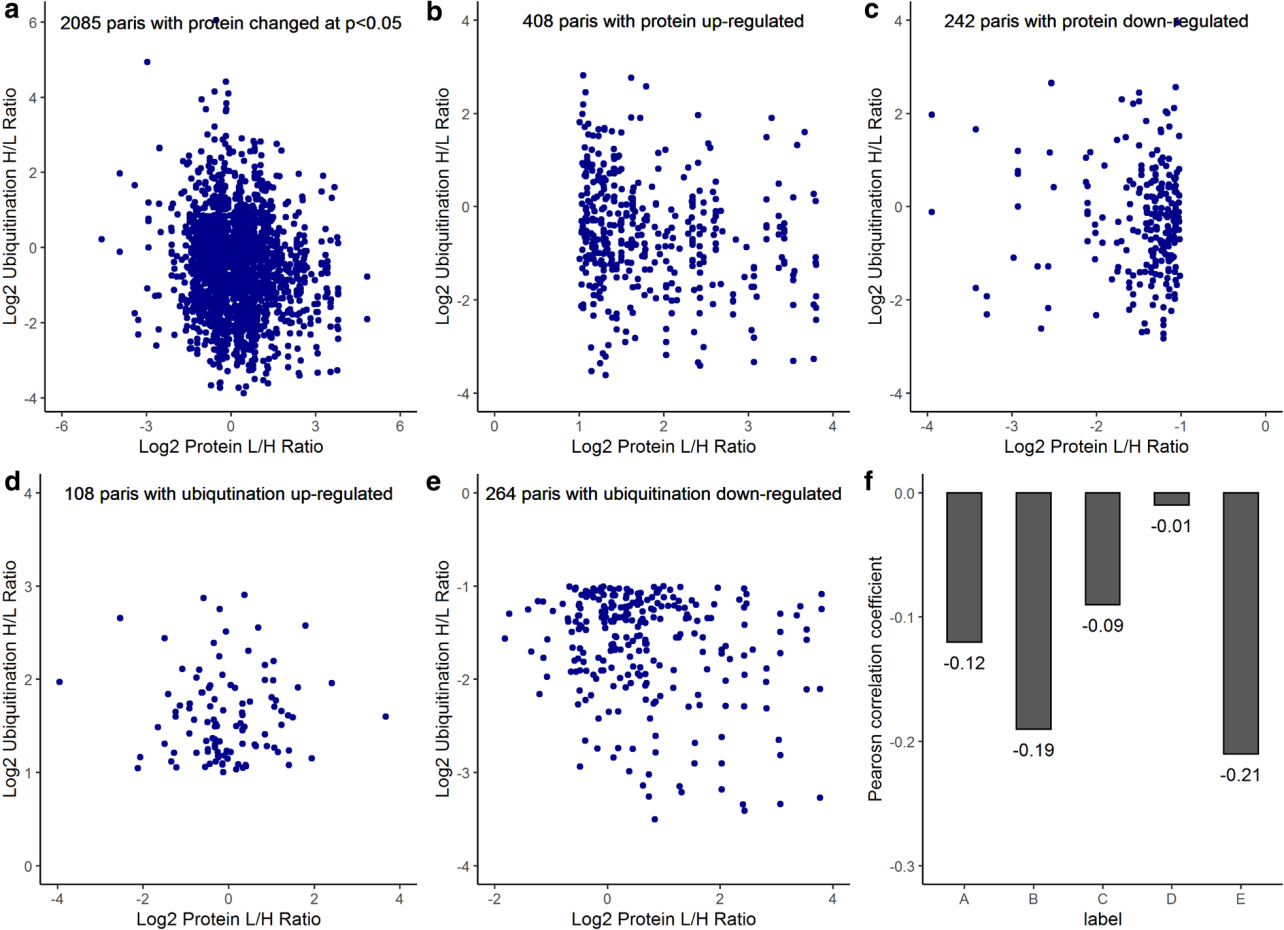
The Correlation between the Global Proteome and Ubiquitylome. **a** Correlation between proteome and ubiquitylome fold changes upon HBV integration for all ubiquitination-protein pairs in HepG2.2.15 cells. **b** Significantly up-regulated proteins. **c** Significantly down-regulated proteins. **d** Significantly up-regulated ubiquitination. **e** Significantly down-regulated ubiquitination. **f** Pearson correlations comparisons for **a**–**e**, are shown

The interaction between the whole proteome and ubiquitylome in response to HBV integration was analyzed based on the quantitative results obtained in this study. We found 1166 quantified proteins possess 2612 ubiquitinated Lys sites that underwent ubiquitination (Fig. 7a). Of the 1166 quantified proteins, 120 proteins were down-regulated and 210 were up-regulated.

The Pearson’s correlation coefficient was calculated as − 0.12 of all significantly ubiquitinated proteins (Fig. 7a, f). Therefore, the global proteome and ubiquitylome were negatively correlated, which implies that, to a certain extent, the changing pattern of the proteome was opposite that of the ubiquitylome in response to HBV. Confined the analysis to pairs of up-regulated proteins and pairs of down-regulated proteins increased the correlation (r = − 0.19 and − 0.09, respectively (Fig. 7b, c, f). For ubiquitination/protein pairs with significantly up-regulated and significantly down-regulated ubiquitination, two weak negative correlations were observed (r = − 0.01 and − 0.21, respectively; Fig. 7d–f). These results suggested that proteome expression levels were negatively regulated by ubiquitination.

### HBV induced changes in membrane and vesicular transport proteins

**Fig. 8:**
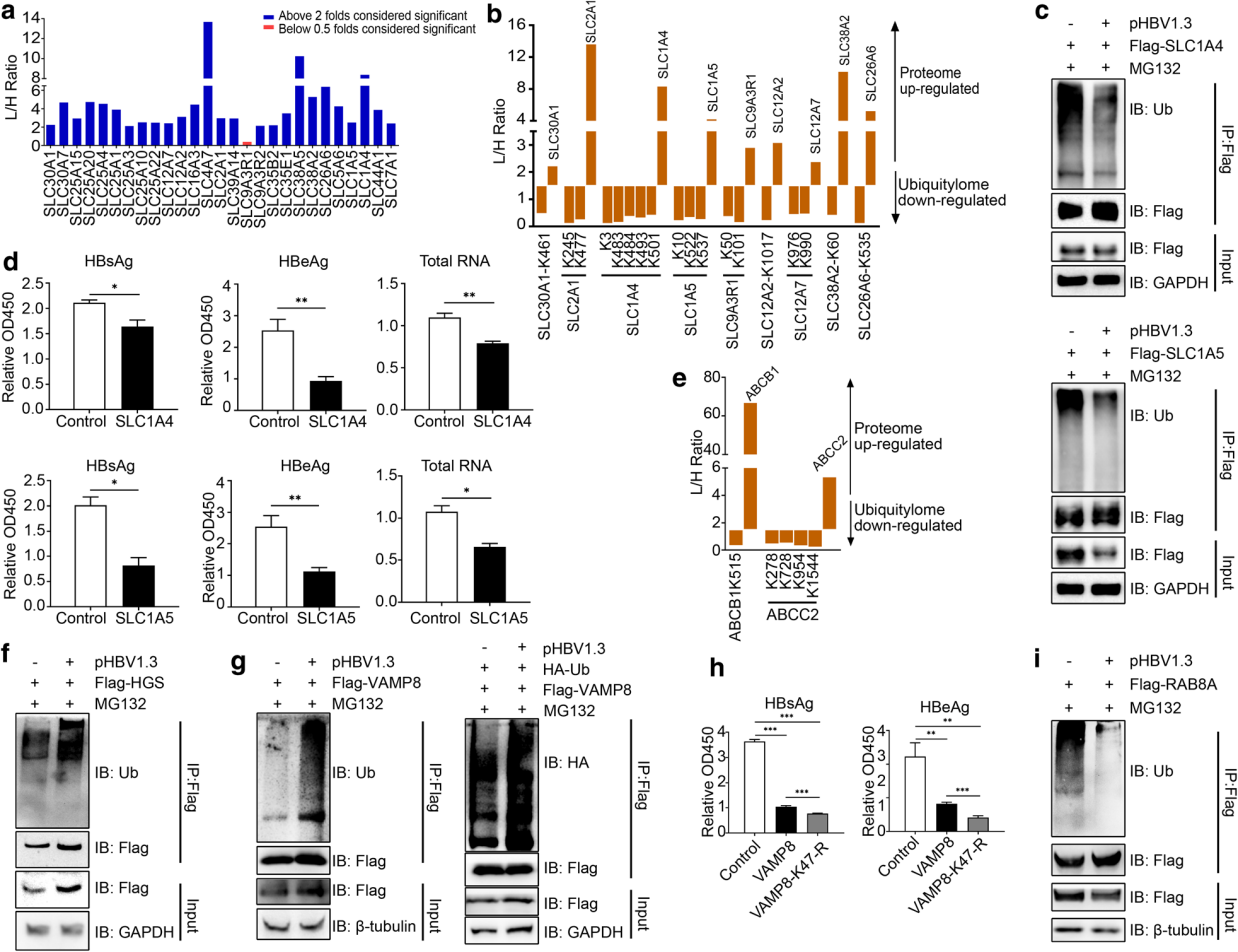
Modifications in membrane and vesicular transport proteins upon HBV integration. **a**, **b**, **e** L/H ratios for selected regulated Kub-sites & proteins from three biological replicates in HepG2.2.15 cells. **c** Co-immunoprecipitation and immunoblot analysis of extracts of Huh7 cells transfected with various combinations of plasmid encoding FLAG-tagged SLC1A4, SLC1A5 and Hepatitis B virus (HBV) 1.3-fold genome plasmid (pHBV1.3) and treated with MG132, are shown. **d** The levels of the secreted HBeAg and HBsAg were determined by ELISA from the cell culture supernatants samples co-transfected (1:1) with pHBV1.3 and flag-tagged SLC1A4 and SLC1A5, are shown. **f** Co-immunoprecipitation and immunoblot analysis of extracts of Huh7 cells transfected with plasmid encoding FLAG tagged-HGS and Hepatitis B virus (HBV) 1.3-fold genome plasmid (pHBV1.3) and treated with MG132, are shown. **g**, **h** Co-immunoprecipitation and immunoblot analysis of extracts of Huh7 cells transfected with FLAG-tagged VAMP8 and HA-tagged wild type ubiquitin, Hepatitis B virus (HBV) 1.3-fold genome plasmid (pHBV1.3) and treated with MG132, are shown. The levels of the secreted HBeAg and HBsAg were determined by ELISA from the cell culture supernatants samples co-transfected (1:1) with pHBV1.3 and flag-tagged VAMP8 and its K-47-R lysine to Arginine mutant. **i** Co-immunoprecipitation and immunoblot analysis of extracts of Huh7 cells transfected with FLAG-tagged RAB8A and Hepatitis B virus (HBV) 1.3-fold genome plasmid (pHBV1.3) and treated with MG132, are shown

The solute carrier (SLC) and ATP-binding cassette (ABC) are two large superfamilies of membrane transport proteins. In this study, in the proteome 27 Solute carrier (SLC) and 8 ATP-binding cassette (ABC) transporter proteins were differentially expressed (Fig. 8a). Among the SLC family, 26 members were up-regulated except for SLC9A3R2, including members of zinc transporter (SLC30A1, SLC30A7), mitochondrial carrier (SLC25A15, SLC25A20, SLC25A4, SLC25A1, SLC25A3, SLC25A10, SLC25A22), electroneutral cation-Cl co-transporter (SLC12A7, SLC12A2), mono-carboxylate transporter (SLC16A3), bicarbonate transporter (SLC4A7), facilitative GLUT transporter (SLC2A1, the most up-regulated up to 13.67-folds), metal ion transporter (SLC39A14), sodium/hydrogen exchanger (SLC9A3R1, SLC9A3R2), nucleotide–sugar transporter (SLC35B2, SLC35E1), Na(+)-coupled neutral amino acids transporter (SLC38A5, SLC38A2), multifunctional anion exchanger (SLC26A6), sodium-glucose co-transporter (SLC5A6), choline transporter (SLC44A1), cationic amino acid transporter (SLC7A1) and neutral amino acid transporter (SLC1A5, SLC1A4). Importantly, in the ubiquitylome we found that the ubiquitination of 18 ubiquitinated Lys sites in 9 SLC proteins SLC30A1 (K461), SLC2A1 (K245, K477), SLC1A4 (K3, K483, K484, K493, K501), SLC1A5 (K10, K522, K537), SLC9A3R1 (K50, K101), SLC12A2 (K1017), SLC12A7 (K976, K990), SLC38A2 (K60) and SLC26A6 (K535) were significantly down-regulated (Fig. 8b). Consistent with our quantitative data, we found that HBV substantially reduced the ubiquitination of exogenous SLC1A4 and SLC1A5 (Fig. 8c). Notably, over-expression of SLC1A5 and SLC1A4 inhibited HBV propagation (Fig. 8d). In addition, we found that HBV induced extraordinarily up-regulation of ATP-binding cassette (ABC) superfamily efflux transporter proteins, ABCB1 (up to 67-folds) and ABCC2 (up to 5.36-folds), on the other hand, down-regulated their ubiquitination level at lysine positions K515 and K278, K728, K954, K1544 respectively (Fig. 8e). Overexpression of ABCC2 was previously reported in pancreatic cancer (Cervenkova et al. 2019). However, ABCC2 was shown to be down-regulated by hepatitis B virus core protein in the combined proteomics and metabolomics approach in hepatocellular carcinoma cells [19]. The expression levels of ABCs regulate the drug concentration inside the cells and up-regulation of ABCs is associated with multi-drug resistance reported in many cancers, HIV and hepatitis B infected patients [31]. Taken together, it suggests that HBV negatively regulates ubiquitination of solute carrier (SLC) and ATP-binding cassette (ABC) proteins.

A large number of enveloped viruses utilize ESCRT pathway for budding from cells. Endosomal Sorting Complex ESCRT-0 consists of three cellular factors HGS, STAM1 and STAM2. Over-expression of wild type HGS protein suppresses HBV DNA synthesis [32]. In our data, HGS was significantly ubiquitinated at lysine positions K86, K226 and K251 as shown in Fig. 5B**.** Our immunoblot analysis confirmed HBV-induced ubiquitination of exogenous HGS (Fig. 8f). Moreover, SNARE associated vesicular transport protein VAMP8 was also found to be substantially ubiquitinated at lysine position K47 (shown in Fig. 5b), and its ubiquitination up-regulation was verified (Fig. 8g). VAMP8 was shown to be down-regulated in multi-omics analyses in response to HBV invasion [19]. It interacts with SNAP29 to modulate HBV replication and its silencing significantly increased the virion production [33]. Consistently, over-expression of VAMP8 significantly suppressed the HBV protein expression, but this inhibition was independent of its K47 lysine ubiquitination (Fig. 8h). The ubiquitination modifications of VAMP8 and HGS in response to HBV infection have not been reported previously, which might be strategy utilized by HBV to silence their protein expression via K48-mediated proteasomal degradation. In addition, ubiquitination of RAB8A which is involved in intracellular membrane trafficking was highly down-regulated consistent with our quantitative findings (Fig. 8i). As a Rab protein, Rab8b is required for HCV [34] and Hantavirus [35] particle release. Further research is needed to understand the exact functional output of these modifications and whether they affect transport proteins turnover or intracellular transport.

### Host E3s and DUBs proteome and ubiquitylome dynamics upon HBV integration

**Fig. 9:**
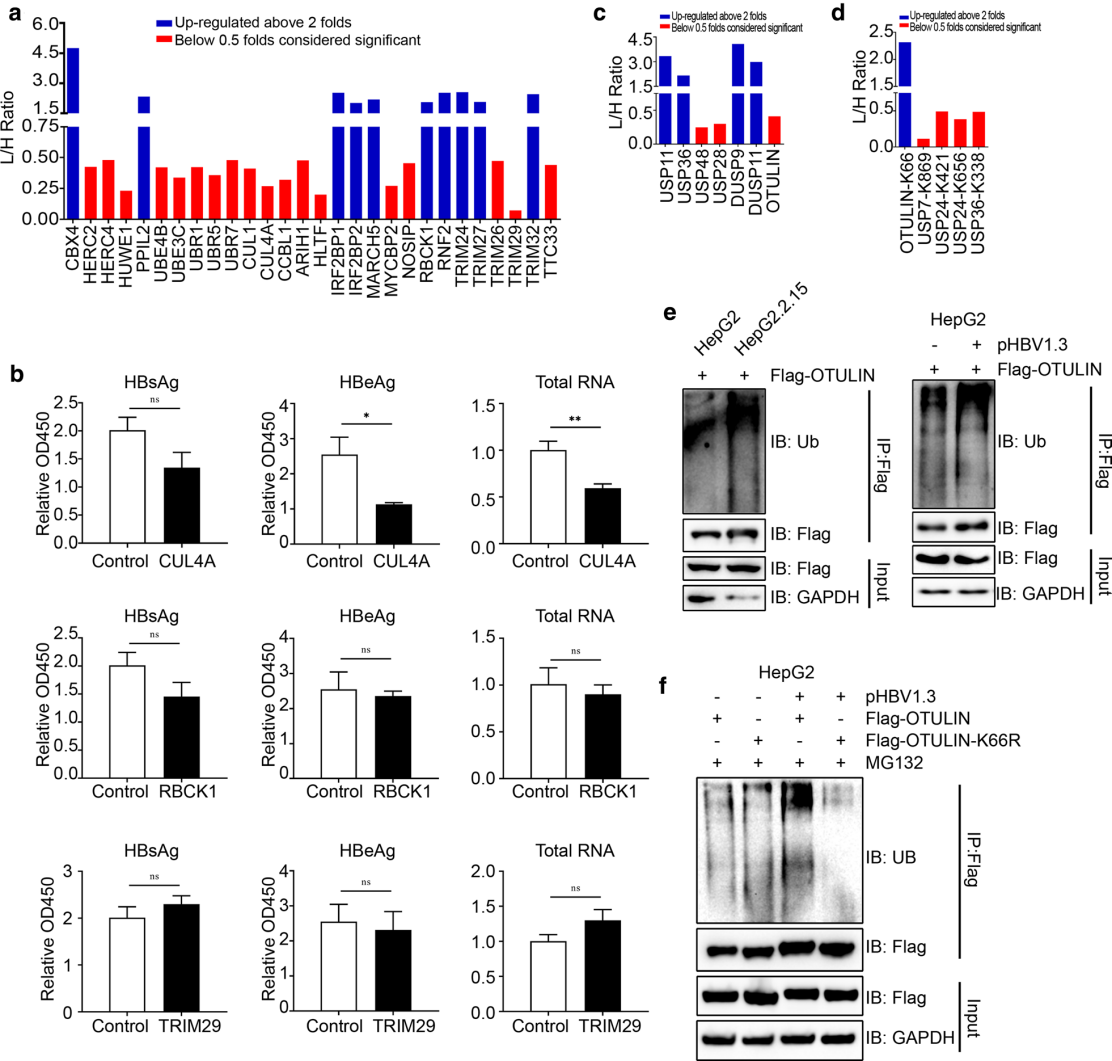
HBV induced changes in the regulation Host heterogeneous nuclear ribonucleoproteins (hnRNPs), E3s and DUBs. **a** L/H ratios for selected regulated proteins from three biological replicates in HepG2.2.15 cells. **b** The levels of the secreted HBeAg and HBsAg were determined by ELISA and HBV total RNA by RT-PCR from samples co-transfected (1:1) with pHBV1.3 and indicated plasmids. **c** L/H ratios for selected regulated proteins from three biological replicates in HepG2.2.15 cells. **d** L/H ratios for selected regulated Kub-sites & proteins from three biological replicates in HepG2.2.15 cells. **e**, **f** Co-immunoprecipitation and immunoblot analysis of extracts of HepG2.2.15 & HepG2 cells transfected with plasmid encoding Flag-OTULIN, Flag-OTULIN-K66-R and Hepatitis B virus (HBV) 1.3-fold genome plasmid (pHBV1.3) and treated with MG132, are shown

In our results, we found 28 E3 ubiquitin ligases were differentially expressed, among which 67% were down-regulated in response to HBV integration (Fig. 9a; Table 1). We evaluated the effects of ectopic expression of TRIM29, RBCK1, RAD18 and CUL4A upon HBV replication. Expression of CUL4A significantly reduces the amounts of secreted HBsAg, HBeAg and HBV total RNA, but our study did not reveal any significant effect of TRIM29, RAD18 and RBCK1on HBV replication (Fig. 9b). Considering the limited number of HBV proteins, these cellular E3s are more possibly find their cognate substrates in host proteins.

**Table 1.** E3 ubiquitin ligases and deubiquitinases found to be significantly altered in response to HBV integration

Protein accession	Protein description	Gene name	L/H ratio	Regulated type
E3 ubiquitin ligases				
O00257	E3 SUMO-protein ligase CBX4	CBX4	4.756	Up
O95714	E3 ubiquitin-protein ligase HERC2	HERC2	0.425	Down
Q5GLZ8	Probable E3 ubiquitin-protein ligase HERC4	HERC4	0.480	Down
Q7Z6Z7	E3 ubiquitin-protein ligase HUWE1	HUWE1	0.232	Down
Q13356	Peptidyl-prolyl cis–trans isomerase-like 2	PPIL2	2.341	Up
O95155	Ubiquitin conjugation factor E4 B	UBE4B	0.421	Down
Q15386	Ubiquitin-protein ligase E3C	UBE3C	0.337	Down
Q8IWV7	E3 ubiquitin-protein ligase UBR1	UBR1	0.422	Down
O95071	E3 ubiquitin-protein ligase UBR5	UBR5	0.357	Down
Q8N806	Putative E3 ubiquitin-protein ligase UBR7	UBR7	0.479	Down
Q13616	Cullin-1	CUL1	0.410	Down
Q13619	Cullin-4A	CUL4A	0.267	Down
Q16773	Kynurenine–oxoglutarate transaminase 1	CCBL1	0.319	Down
Q9Y4X5	E3 ubiquitin-protein ligase ARIH1	ARIH1	0.476	Down
Q14527	Helicase-like transcription factor	HLTF	0.200	Down
Q8IU81	Interferon regulatory factor 2-binding protein 1	IRF2BP1	2.531	Up
Q7Z5L9	Interferon regulatory factor 2-binding protein 2	IRF2BP2	2.016	Up
Q9NX47	E3 ubiquitin-protein ligase MARCH5	MARCH5	2.189	Up
O75592	E3 ubiquitin-protein ligase MYCBP2	MYCBP2	0.270	Down
Q9Y314	Nitric oxide synthase-interacting protein	NOSIP	0.454	Down
Q9BYM8	RanBP-type and C3HC4-type zinc finger-containing protein 1	RBCK1	2.062	Up
Q99496	E3 ubiquitin-protein ligase RING2	RNF2	2.531	Up
O15164	Transcription intermediary factor 1-alpha	TRIM24	2.562	Up
P14373	Zinc finger protein RFP	TRIM27	2.065	Up
Q12899	Tripartite motif-containing protein 26	TRIM26	0.473	Down
Q14134	Tripartite motif-containing protein 29	TRIM29	0.072	Down
Q13049	E3 ubiquitin-protein ligase TRIM32	TRIM32	2.457	Up
Q6PID6	Tetratricopeptide repeat protein 33	TTC33	0.440	Down
Deubiquitinases				
P51784	Ubiquitin carboxyl-terminal hydrolase 11	USP11	3.239	Up
Q9P275	Ubiquitin carboxyl-terminal hydrolase 36	USP36	2.158	Up
Q86UV5	Ubiquitin carboxyl-terminal hydrolase 48	USP48	0.284	Down
Q96RU2	Ubiquitin carboxyl-terminal hydrolase 28	USP28	0.299	Down
Q99956	Dual specificity protein phosphatase 9	DUSP9	4.069	Up
O75319	RNA/RNP complex-1-interacting phosphatase	DUSP11	2.974	Up
Q96BN8	Ubiquitin thioesterase otulin	OTULIN	0.411	Down

7 members of cysteine protease class of deubiquitinating enzymes (DUBs) were found to be differentially expressed, and 4 of them were up-regulated (Fig. 9c, Table 1). Ubiquitination of USP7 (K869), USP24 (K421, K656) and USP36 (K338) were down-regulated. Whereas, ubiquitination of OTULIN (K66) was significantly up-regulated, resulting in a decline in its protein level (Fig. 9d). Noticeably, HBV-induced ubiquitination of exogenous OTULIN was confirmed by immunoblotting (Fig. 9e). To further address the relevance of K66 lysine site for the up-regulation of OTULIN’s ubiquitination, we compared ubiquitination of wild-type and K66R mutant of OTULIN upon HBV replication. Single-mutant variants displayed a significant reduction in ubiquitination compared to wild-type OTULIN (Fig. 9f), implying that K66R might be a key ubiquitination site for K48-linked poly-ubiquitination which could lead to OTULIN degradation. Strategies may be adopted by HBV to regulate E3 ligases and DUBs of host cells for ubiquitination and regulation of various host and viral proteins.

## Discussion

Owing to its functional and regulatory diversity and its key roles in regulating various protein activities and cellular events, ubiquitin system becomes particularly attractive target for viruses to manipulate to favor their propagation. On the other hand, host cells could also target virus proteins for degradation by ubiquitin–proteasome pathway to fight against virus invasion. Although considerable data is available regarding the HBV-mediated alterations in the transcriptome [16], the proteome of exosomes [20, 21], the proteome of host cell lipid rafts [29], combined proteomics and metabolomics analyses induced by Hepatitis B virus core protein, the modifications in host ubiquitylome and proteome induced by HBV were still unexplored. In this study, changes in host global ubiquitylome and proteome were evaluated with or without HBV integration in HepG2.2.15 and HepG2 cell lines, respectively. The HepG2.2.15 cell line is stably transfected with the complete HBV genome, which can express HBV RNA and viral proteins, and produce infectious virus-like particles. HepG2.2.15 cell line cannot be used to study the process of HBV infection. However, the HBV infection efficiency of HepG2-NTCP cell line is too low to satisfy the requirement for ubiquitylome difference analysis; and HLCZ01 cells used to study the entire life cycle of HBV are unable to be cultivated for enough generations to reach a number of 10^7^–10^8^ cells necessary for our research. Furthermore, cells from patient tissues can neither be cultivated for enough generations and labeled in culture. Considering that HBV-induced HCC occurs not at the initial stage of virus infection, but rather gradually develops along the path of “HBV infection-liver cirrhosis-HCC”, thus HepG2.2.15 becomes an ideal tool for us to study the regulatory role of ubiquitination on HBV replication, immune tolerance and HCC progression. It should be aware that HBV may inhibit the proliferation ability of HepG2.2.15 cells by regulating their cell cycle, thus the observed changes of the proteome and ubiquitylome in HepG2.2.15 cells still need futher investigation through in-depth experiments to examine whether they are truly HBV-induced.

The global analysis of HBV-mediated host proteome and ubiquitylome change in HepG2.2.15 cells demonstrated that HBV integration significantly altered the host ubiquitylome and proteome. In total, 7188 proteins were identified using quantitative proteomics and nearly 19% of all identified proteins being over twofold regulated. Moreover, our study further identified 3798 lysine ubiquitination sites in 1746 host proteins using quantitative proteomics coupled to tryptic Ub remnant-based enrichment methodology, corresponding to HBV induced changes in a number of diverse cellular processes including metabolism and cell cycle, transport and vesicle trafficking, and in particularly E3s and DUBs enzymes.

The enrichment of metabolic pathways in global proteome indicated a potential impact of HBV on cellular functions related to DNA replication, mitochondria, membrane and metabolism, prominently NADH dehydrogenase ubiquinone, ATP synthase, cytochrome oxidase complex proteins and ion transmembrane transporters. The enhanced TCA cycle is associated with viral envelopment, enlargement of the nucleus, and of vesicular bodies of the virus invaded cells [36]. HBV also enhances proteins involved in the metabolism of cholesterol and biosynthesis of bile acids. HBc promoted the expression of metabolic enzymes and the secretion of metabolites in HCC cells [19]. In the last decade, several studies have concluded that the progression of cancer involves major alterations in cell metabolism and metabolic alterations induced by HBV [19, 37]. Aldolase A (ALDOA), a key glycolytic enzyme has been reported to be highly expressed in several cancers including pancreatic cancer, clear cell renal carcinoma, cervical, gastric cancer and most recently in HCC. Our results are consistent with previously published research, moreover, make us know more about the role of ubiquitination on host protein alteration upon HBV integration.

To validate the accuracy of our results, we have picked 16 genes to experimentally confirm their ubiquitination changes after HBV transfection, and 62.5% (10 proteins) gave consistent results with UbiScan. In this paper, we first report and demonstrated ubiquitination up-regulation of TOLLIP, HGS, OTULIN and VAMP8, as well as ubiquitination down-regulation of VAMP3, VDAC2, LYN, SLC1A4, SLC1A5, DNAJB6 and RAB8A after HBV transfection. It has been reported that VAMP3 interacts with Human Herpesvirus 6 (HHV-6) via its glycoprotein M and its expression gradually increases during the late phase of virus invasion [38]. In a similar way, VAMP3 was also found to be interacted with bunyavirus Uukuniemi virus (UUKV) and required for its late penetration [39]. VDACs play an important role in mitochondrial dysfunction and reactive oxygen species (ROS) signaling. VDAC3 was found to interact with HBx [40]. Furthermore, we experimentally confirmed ubiquitination of another protein DNAJB6 and its ubiquitination was down-regulated which was in consistent with our UbiScan results. Over-expression of DNAJB6 could significantly reduced the amounts of secreted HBsAg and HBeAg. Similar to our results, DNAJB6 proteins particularly enhanced the degradation of core and HBx proteins of HBV [41]. Taken together, Ubiscan method could provide us relatively reliable information regarding the global proteome and ubiquitylome of cells, but we could not consider it as a complete and absolute method. Further verifications are needed before making any final conclusion.

By comparing the quantitative results of proteome and ubiquitylome, we revealed that the expression levels of proteins in global proteome are negatively correlated to the ubiquitination levels indicated by Pearson's correlation coefficient − 0.12. It means that the increased ubiquitination level of a specific protein induced by HBV decreased the protein level correspondingly. It is generally consistent with the protein-degradation function of ubiquitination. Interestingly, we found that 67% of 28 differentially expressed E3 ubiquitin ligases were down-regulated and 57% of 7 distinguishably expressed deubiquitinases were up-regulated (Table 1). These distinct modifications in protein expression of E3s and DUBs were in accordance with ubiquitination down-regulation of 71% ubiquitinated Lys sites of twofold-modified lysine ubiquitylome in our quantitative data. USP15 was found to directly interact with HBx and increased its protein level in a dose-dependent manner [42]. In addition, other polyubiquitination which we didn’t address in this paper, also has many other roles in protein modification, such as altering biochemical properties and subcellular protein localization [11]. K27- or K63-linked ubiquitination mediated by various E3 ubiquitin ligases, such as TRIM32, AMFR and INSIG1, is essential for full activation of STING [43]. TRIM22 inhibits the transcriptional activities of the hepatitis B virus (HBV) core promoter and HIV-1 LTR [44]. Previously, it is reported that over-expression of TRIM5, 6, 11, 14, 25, 26, 31 and 41 efficiently reduced the amounts of secreted HBsAg and HBeAg and total RNA [45]. In addition, USP13 removes K27-linked polyubiquitinchains from STING then decreases the antiviral immune response against DNA viruses by disrupting the recruitment of TBK1, whereas USP7 removes K48-linked polyubiquitination from TRIM27 and promotes the degradation of TBK1. Similarly, USP21 deubiquitinates the K27/63-linked polyubiquitin chain on STING, thereby leading to reduced production of type I IFN [43]. This might be a manipulation by HBV to develop means to enhance or inhibit ubiquitylation of host-specific substrates, depending on its needs. Based on our results that HBV induced massive changes in protein level and protein ubiquitination and 4 types of conserved ubiquitination motifs were observed in 987 unique sites, we speculate that HBV may regulate the levels of enormous host protein by altering their ubiquitination through identified E3s and DUBs (Table 1). Further intensive research is required to verify this hypothesis.

Our results also showed that HBV substantially induced changes in protein levels of 27 solute carrier (SLC) and 8 ATP-binding cassette (ABC) membrane transport proteins. SLC transporters are primarily uptake transporters involved in amino acids, sugars and nucleotides, whereas ABC superfamily is primarily effluxing transporters by using ATP hydrolysis [46]. SLC30A1 and SLC35A1 represent novel host factors that affected the induction of apoptosis upon VSV virus infection [47]. SLC15A3 contributes to antiviral innate immune responses against Herpes Simplex Virus-1 [48]. Up-regulation of ABCA1 gene expression and its cholesterol efflux function impairs HCV infection and decreases levels of virus production [49]. HCV up-regulated both mRNA and protein expression levels of SLC3A2 and exploits for its cellular entry [50]. SLC transporters have been reported in many cancers and further research is in progress to exploit their potentials as prognostic indicators and therapeutic targets [51]. In pancreatic cancer patients, overexpression of ABCC2 along with SLC22A3 in a combination was detected [52]. Astonishingly, our research showed that the levels of considerable number of SLC and ABC transporter proteins were significantly affected by HBV, providing a promising direction for future research to target the role of SLC and ABC proteins in HBV-mediated HCC.

Heterogeneous nuclear ribonucleoproteins (hnRNPs), a large family of RNA-binding proteins (RBPs), contribute a significant role in alternative splicing, mRNA stabilization, transcriptional and translational regulation [53]. In our data, remarkably we found the ubiquitination of 18 ubiquitinated Lys sites in 11 members of hnRNPs family was down-regulated. Over-expression of hnRNP K augmented HBV replication, while gene silencing of endogenous hnRNP K carried out by small interfering RNAs resulted in a significant reduction of HBV viral load [54]. hnRNP K is a potential tissue biomarker, either alone or in combination with serum AFP, for detection of early HCC [55]. hnRNP L and NF90 can interact with the 5′-terminal untranslated RNA of Hepatitis C Virus and promote viral replication [56]. In our results, hnRNP K, M and L proteins all showed reduced ubiquitination modification upon HBV integration. hnRNP M and hnRNP K are essential factors in tumor development and progression [57]. In recent studies, significantly enhanced expression of hnRNP A1 has been reported to be associated with HBV-mediated HCC [58]. The prominent ubiquitination down-regulation induced by HBV in hnRNP family members implies possible roles of their ubiquitination in HBV-mediated HCC. Further extensive functional and mechanism studies are certainly needed to address such roles and their possible applications in design of novel therapeutic and prognostic targets for HBV-mediated HCC.

Ubiquitination has been found to be involved in regulation of virus infection, immune regulation and tumor progression. By exploring the regulatory role of ubiquitination system during host immune response, cell metabolism, cell proliferation and liver carcinogenesis after HBV integration, we are possible to explore the application prospect of ubiquitin system during prevention and treatment of HBV-associated HCC. Carfilzomib (PR-171), as an inhibitor of the ubiquitin–proteasome system, has been approved by the FDA for the treatment of multiple myeloma and clinical trials for the treatment of Hematologic Malignancies [59, 60]. Aiming at certain ubiquitinases or substrates which play important roles during HBV-mediated HCC, more specific inhibitors could be designed as novel drugs for possible prevention and treatment of HBV-mediated HCC.

## Conclusions

Overall, in this study by employing highly sensitive SILAC-based quantification analysis of host ubiquitylome and proteome using HepG2.2.15, a cell line stably producing HBV virus, we demonstrated that HBV significantly modify the host ubiquitylome and proteome. The protein level of approximately 19% of all identified proteins was changed over twofold involved in various biological processes particularly in cellular respiration, membrane transport and ubiquitination were highly up-regulated whereas proteins involved in DNA synthesis were down-regulated in response to HBV integration, render a cellular environment which favors HBV persistent replication within the host liver. Based on our findings, it is reasonable to assume that it provides a valuable resource of information related to the complex network of host-virus interactions and the impact of HBV-mediated changes to normal hepatocyte physiology on viral replication.

## Supplementary Information

**Additional file 1: Figure S1.** Functional enrichment-based clustering analysis for the quantified proteome. (A) The subcellular location of up-regulated. (B) The subcellular location of down-regulated. (C) GO-based enrichment analysis of up-regulated and down-regulated proteins. (D) Heatmap representation of cellular component analysis. (E) Heatmap representation of biological process analysis. (F) Heatmap representation of molecular function analysis.

**Additional file 2: Figure S2.** KEEG pathway, protein domain and protein complex analysis for the quantified proteome. (A) KEEG pathway analysis of up-regulated and down-regulated proteins. (B–D) L/H ratios plots for selected regulated proteins of KEEG pathway, protein domain and protein complex analysis from three biological replicates in HepG2.2.15 cells.

**Additional file 3: Figure S3.** Functional enrichment-based clustering analysis for the quantified ubiquitylome in response HBV integration. (A) Venn diagrams of Kub-sites and proteins in HepG2.2.15. Kub-sites numbers are indicated. (B) GO-based enrichment analysis of up-regulated and down-regulated Kub-sites. (C) Heatmap representation of biological process analysis. (D) L/H ratios for selected regulated Kub-sites & proteins from three biological replicates in HepG2.2.15 cells. (E) Heatmap representation of molecular function analysis.

**Additional file 4: Figure S4.** Protein complex and protein domain enrichment analysis for the quantified ubiquitylome. (A) Protein complex enrichment analysis of up-and down-regulated Kub sites and their Heatmap representation. (B) Protein domain enrichment analysis of up-and down-regulated Kub sites and their Heatmap representation.

**Additional file 5: Table S1.** Total identified and quantified proteins in response to HBV integration in HepG2.2.15 cells.

## Data Availability

The datasets used and/or analyzed during the current study are available from the corresponding author on reasonable request.

## Abbreviations

HBV: Hepatitis B virus
SILAC: Stable Isotope Labeling with Amino Acids in Cell Culture
HCC: Hepatocellular carcinoma
MS: Mass spectrometry
ELISA: Enzyme-linked immunosorbent assay
GAPDH: Glyceraldehyde-3-phosphate dehydrogenase
WT: Wild-type
HA: Hemagglutinin
HBeAg: Hepatitis B e antigen
HBsAg: Hepatitis B surface antigen
IP: Immunoprecipitation
mRNA: Messenger RNA
qRT-PCR: Quantitative reverse transcriptase-PCR
VAMP3: Vesicle-associated membrane protein 3
VAMP8: Vesicle-associated membrane protein
RAB8A: Ras-related protein Rab-8A
LYN: Lck/Yes novel tyrosine kinase
VDAC2: Voltage-dependent anion-selective channel protein 2
SLC1A4: Solute Carrier Family 1 Member 4
SLC1A5: Solute Carrier Family 1 Member 5
HGS: Hepatocyte growth factor-regulated tyrosine kinase substrate
ZC3HAV1: Zinc finger CCCH-type antiviral protein 1
EIF4A1: Eukaryotic initiation factor 4A-I
TOLLIP: Toll-interacting protein
BRCC36: Lys-63-specific deubiquitinase BRCC36
OTULIN: Ubiquitin thioesterase otulin
