# Supplementary figures and images for: Global analysis of HBV-mediated host proteome and ubiquitylome change in HepG2.2.15 human hepatoblastoma cell line
Source: Cell Biosci. 2021 Apr 17;11:75. doi: 10.1186/s13578-021-00588-3 (PMC8052555; doi:10.1186/s13578-021-00588-3)

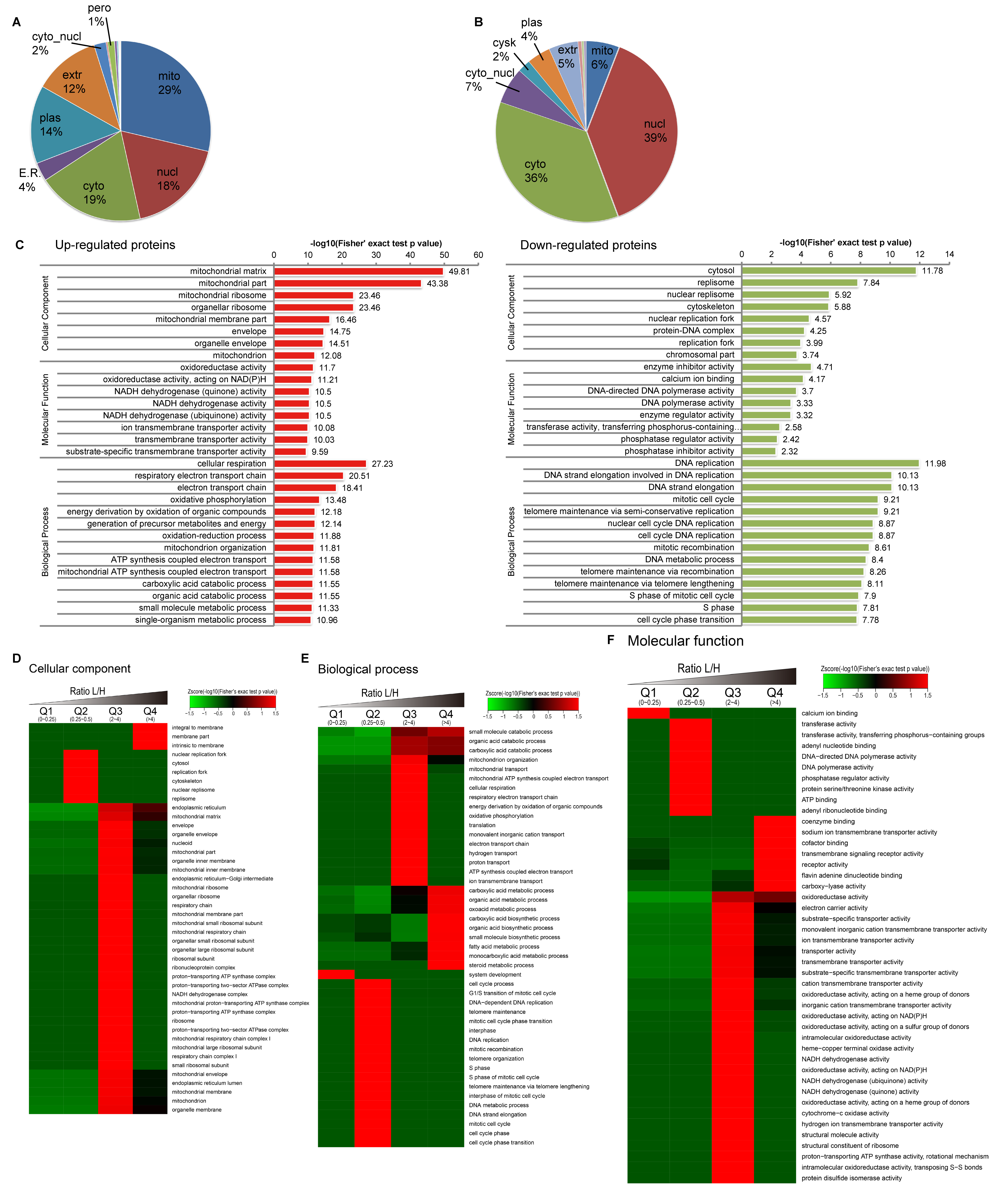

Supplement: Supplementary file 1 — Additional file 1: Figure S1. Functional enrichment-based clustering analysis for the quantified proteome. (A) The subcellular location of up-regulated. (B) The subcellular location of down-regulated. (C) GO-based enrichment analysis of up-regulated and down-regulated proteins. (D) Heatmap representation of cellular component analysis. (E) Heatmap representation of biological process analysis. (F) Heatmap representation of molecular function analysis. [file 13578_2021_588_MOESM1_ESM.tif]

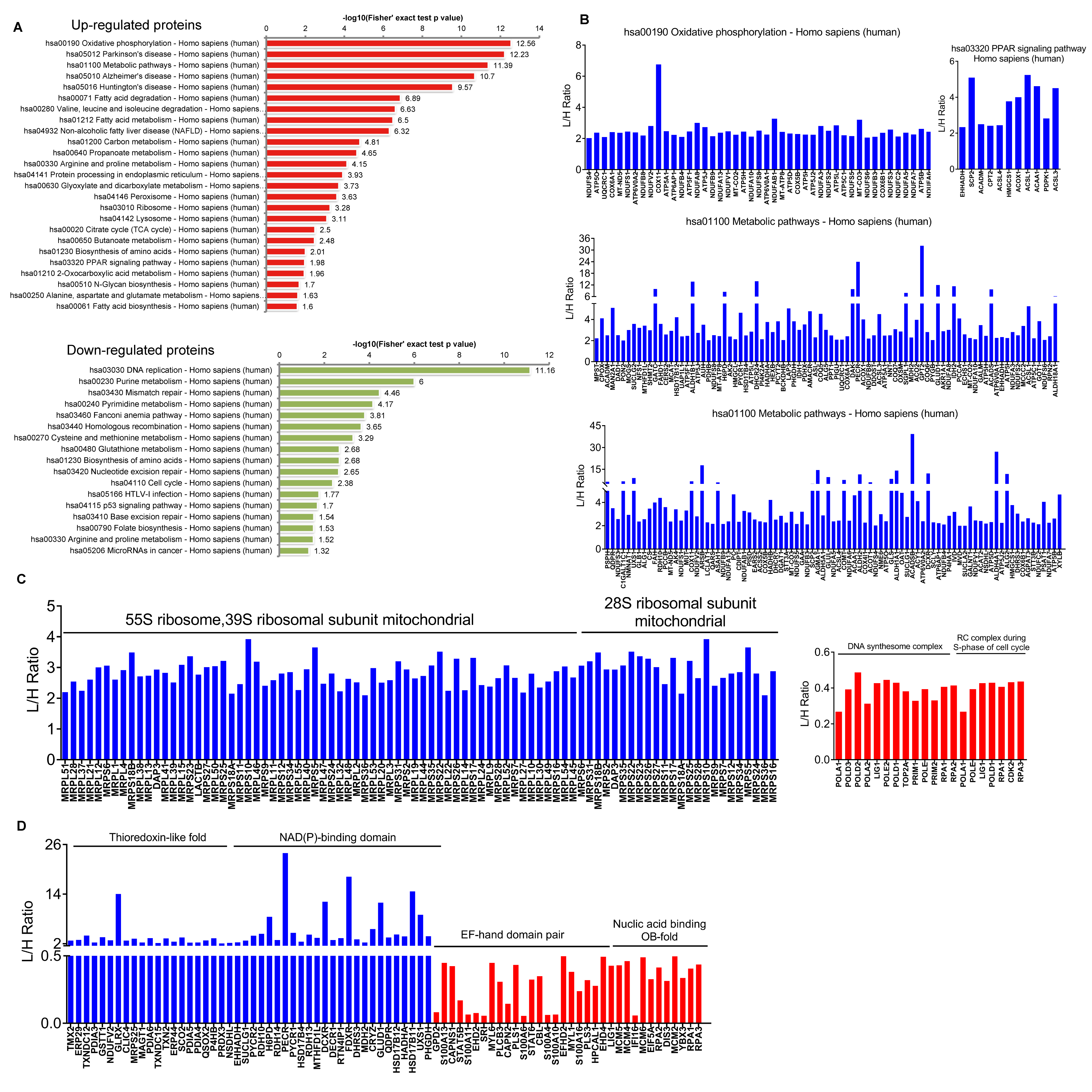

Supplement: Supplementary file 2 — Additional file 2: Figure S2. KEEG pathway, protein domain and protein complex analysis for the quantified proteome. (A) KEEG pathway analysis of up-regulated and down-regulated proteins. (B–D) L/H ratios plots for selected regulated proteins of KEEG pathway, protein domain and protein complex analysis from three biological replicates in HepG2.2.15 cells. [file 13578_2021_588_MOESM2_ESM.tif]

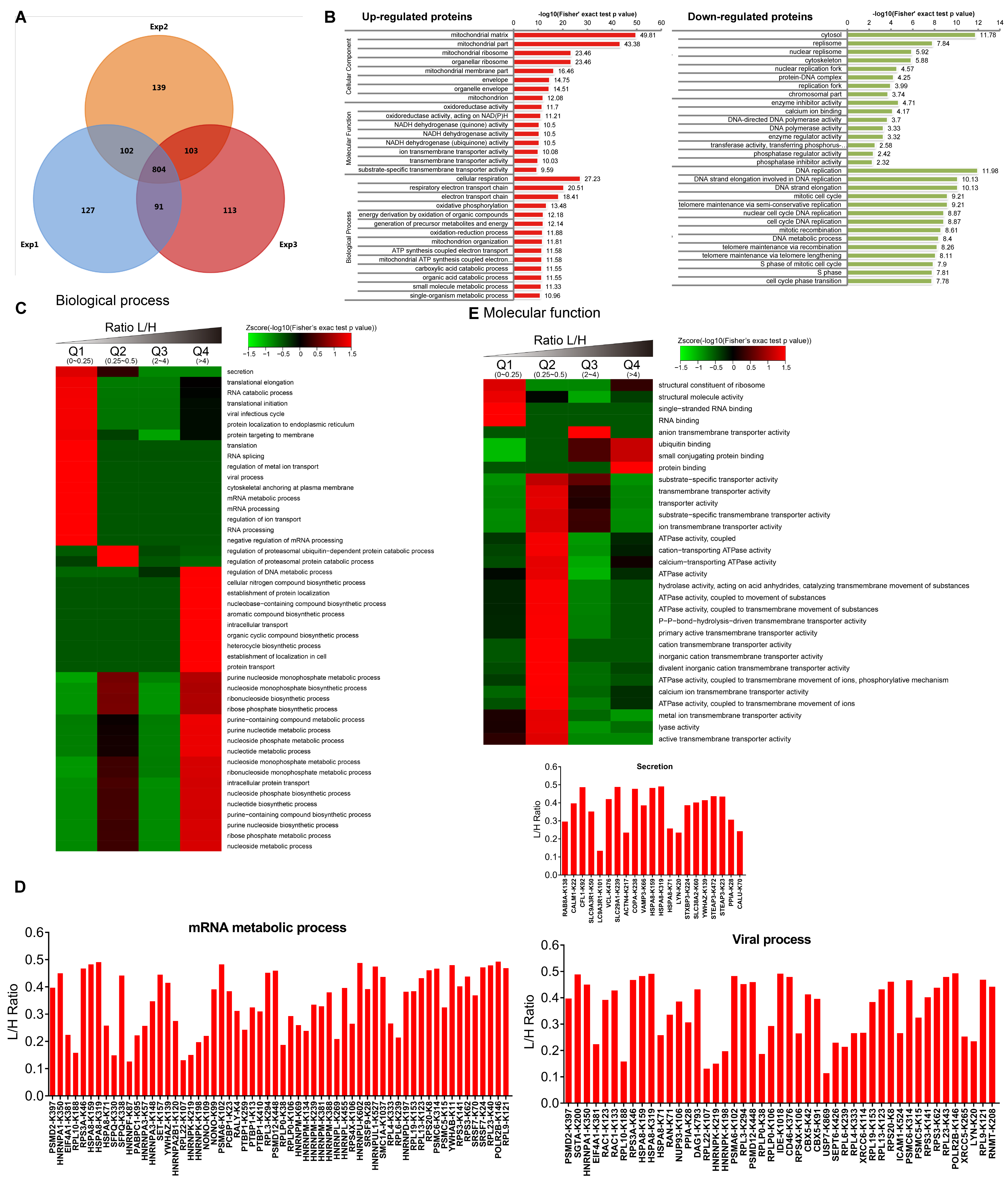

Supplement: Supplementary file 3 — Additional file 3: Figure S3. Functional enrichment-based clustering analysis for the quantified ubiquitylome in response HBV integration. (A) Venn diagrams of Kub-sites and proteins in HepG2.2.15. Kub-sites numbers are indicated. (B) GO-based enrichment analysis of up-regulated and down-regulated Kub-sites. (C) Heatmap representation of biological process analysis. (D) L/H ratios for selected regulated Kub-sites & proteins from three biological replicates in HepG2.2.15 cells. (E) Heatmap representation of molecular function analysis. [file 13578_2021_588_MOESM3_ESM.tif]

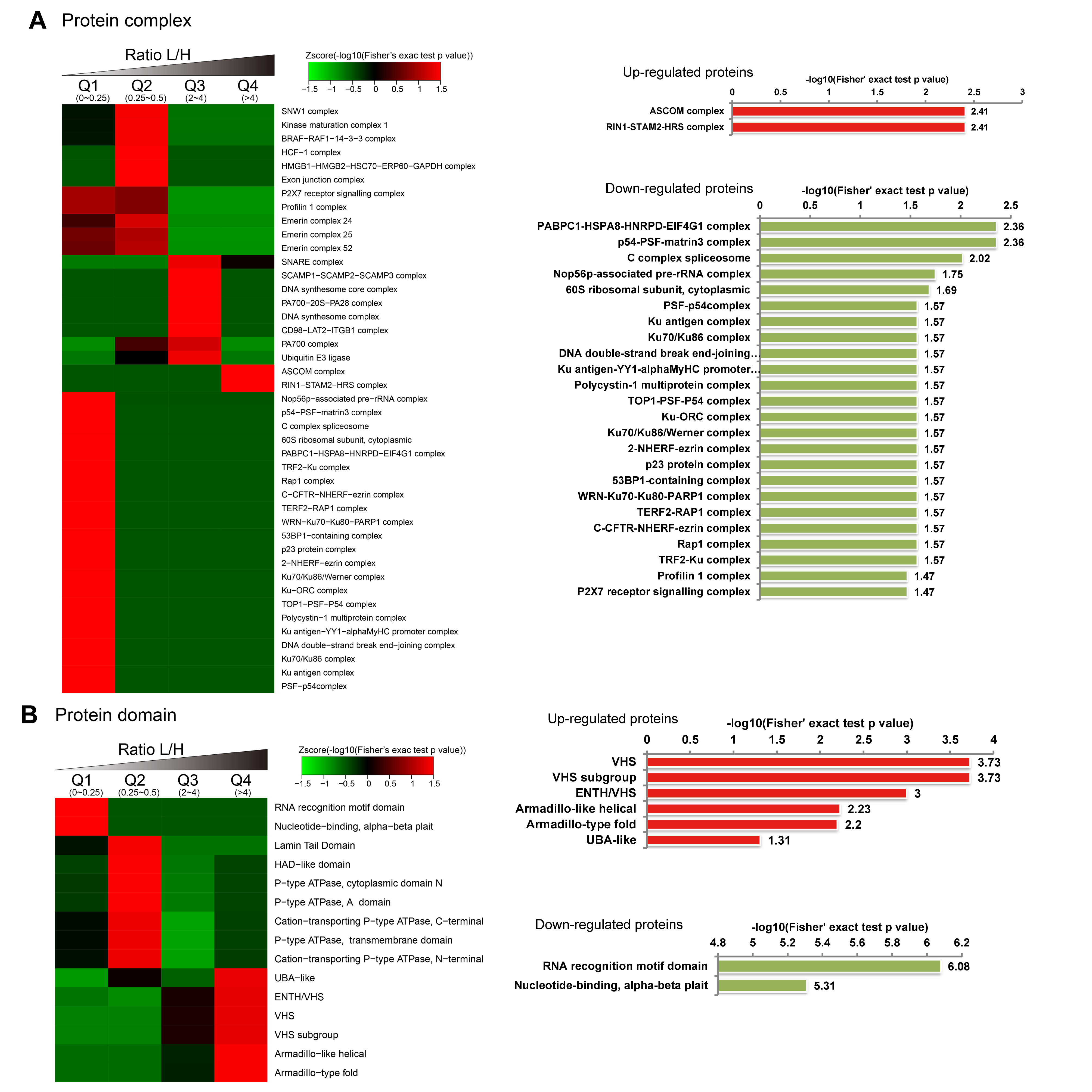

Supplement: Supplementary file 4 — Additional file 4: Figure S4. Protein complex and protein domain enrichment analysis for the quantified ubiquitylome. (A) Protein complex enrichment analysis of up-and down-regulated Kub sites and their Heatmap representation. (B) Protein domain enrichment analysis of up-and down-regulated Kub sites and their Heatmap representation. [file 13578_2021_588_MOESM4_ESM.tif]
